# Lipid profile of circulating placental extracellular vesicles during pregnancy identifies foetal growth restriction risk

**DOI:** 10.1002/jev2.12413

**Published:** 2024-02-14

**Authors:** Miira M. Klemetti, Ante B. V. Pettersson, Aafaque Ahmad Khan, Leonardo Ermini, Tyler R. Porter, Michael L. Litvack, Sruthi Alahari, Stacy Zamudio, Nicholas P. Illsley, Hannes Röst, Martin Post, Isabella Caniggia

**Affiliations:** ^1^ Lunenfeld‐Tanenbaum Research Institute Mount Sinai Hospital Toronto Ontario Canada; ^2^ Department of Obstetrics & Gynecology University of Toronto Toronto Ontario Canada; ^3^ Program in Translational Medicine, Peter Gilgan Centre for Research and Learning Hospital for Sick Children Toronto Ontario Canada; ^4^ Donnelly Centre for Cellular and Biomolecular Research University of Toronto Toronto Canada; ^5^ Placental Research Group LLC Maplewood New Jersey USA; ^6^ Institute of Medical Science University of Toronto Toronto Ontario Canada; ^7^ Department Physiology University of Toronto Toronto Ontario Canada

**Keywords:** lipidomics, placenta, SGA pregnancies, small extracellular vesicles

## Abstract

Small‐for‐gestational age (SGA) neonates exhibit increased perinatal morbidity and mortality, and a greater risk of developing chronic diseases in adulthood. Currently, no effective maternal blood‐based screening methods for determining SGA risk are available. We used a high‐resolution MS/MS^ALL^ shotgun lipidomic approach to explore the lipid profiles of small extracellular vesicles (sEV) released from the placenta into the circulation of pregnant individuals. Samples were acquired from 195 normal and 41 SGA pregnancies. Lipid profiles were determined serially across pregnancy. We identified specific lipid signatures of placental sEVs that define the trajectory of a normal pregnancy and their changes occurring in relation to maternal characteristics (parity and ethnicity) and birthweight centile. We constructed a multivariate model demonstrating that specific lipid features of circulating placental sEVs, particularly during early gestation, are highly predictive of SGA infants. Lipidomic‐based biomarker development promises to improve the early detection of pregnancies at risk of developing SGA, an unmet clinical need in obstetrics.

## Graphical abstract

Distinct lipid signatures of circulating placenta‐derived sEVs define the trajectory of a healthy pregnancy and identify SGA risk in the first trimester of gestation.

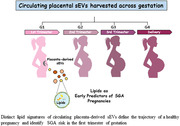

## 1 INTRODUCTION

Low birthweight is a global health problem. Approximately 3%–9% of foetuses in high‐income countries and up to 25% in low‐ and middle‐income countries fail to achieve their genetic growth potential (Lee et al., 2013). In addition to causing neonatal morbidity (Chauhan et al., 2017), inadequate growth in utero contributes to a large proportion of stillbirths (Gardosi et al., 2013; Moraitis et al., 2014), and has adverse consequences for the offspring ranging from neurodevelopmental challenges (Arcangeli et al., 2012) to cardiometabolic morbidity (Crispi et al., 2018). Infants with a birthweight at or below the 10th centile for gestational age are commonly classified as “small‐for‐gestational age” (SGA). While all SGA foetuses do not necessarily display detectable signs of foetal growth restriction (FGR), such as abnormal foetoplacental blood flow and/or reduced growth velocity, SGA with or without FGR is associated with adverse short‐ (Iliodromiti et al., 2017) and long‐term (Figuera et al., 2008) outcomes. Severe growth restriction typically manifests early and leads to iatrogenic preterm birth; however, most SGA foetuses are born at term to mothers with no obvious risk factors and, as such, they are not diagnosed before delivery (Chauhan et al., 2017). This reflects the need to develop tools for early identification of SGA foetuses, enabling closer monitoring as well as individually tailored timing and mode of delivery.

Current ultrasonographic methods provide reasonably accurate estimates of fetal size and enable the assessment of fetoplacental blood flow with Doppler velocimetry. Nevertheless, selective ultrasonography based on risk factors, obstetric complications or serial measurements symphyseal‐fundal height has been shown to identify only ∼20% of SGA foetuses (Sovio et al., 2015). Current evidence does not support the provision of universal late‐pregnancy ultrasound screening due to iatrogenic risks of unnecessary interventions in normal pregnancies (Gaccioli et al., 2018). A British study reported that combined analysis of foetal biometry and abdominal circumference growth velocity in late pregnancy could better identify SGA foetuses at increased risk of neonatal morbidity (Sovio et al., 2015). However, assessment of foetal growth velocity—with at least two ultrasound examinations—is resource‐intensive and is not feasible in all clinical settings. Thus, there is a clear need to develop methods for more accurate identification of the pregnancies that are most likely to benefit from this type of monitoring.

The placenta functions as the primary mediator of foetal nutrient and oxygen transfer and its dysfunction is considered etiological in most SGA cases. Hence, potential biomarkers reflecting impaired placentation and/or placental function have been actively investigated (Conde‐Agudelo et al., 2013; Gaccioli et al., 2018; Heazell et al., 2019). The pro‐angiogenic placental growth factor (PlGF) and anti‐angiogenic soluble fms‐like kinase (sFlt), and their ratio (PlGF/sFlt), have been amongst the most promising and intensely tested biomarkers, but their clinical benefit over ultrasonographic methods has not been demonstrated (Ciobanu et al., 2019). The rapidly expanding field of high‐throughput methodologies has provided new avenues for advancing our understanding of metabolic changes that occur during normal (Lindsay et al., 2015) and pathological pregnancies (Bahado‐Singh et al., 2019; Miranda et al., 2018; Morillon et al., 2021). The availability of unbiased omics tools has also sparked intensive search for novel SGA/FGR biomarkers (Leite et al., 2019; Sovio et al., 2020). A recent metabolomics study on maternal serum suggested that certain metabolite ratios can predict FGR at term, and when used in conjunction with ultrasonic imaging, identify pregnancies in need of growth monitoring and induction of labour at around 36 weeks’ gestation (Sovio et al., 2020). Although intriguing, screening of maternal metabolites is likely to provide mainly indirect reflections of the fetoplacental status. In contrast, analysis of potential biomarkers in extracellular vesicles (EVs) secreted by placental cells into the maternal circulation throughout pregnancy, presents a new, more direct means for obtaining direct information from the fetoplacental unit (Tkach & Théry, 2016). Small EVs (sEV < 200 nm diameter according to guidelines of International Society for Extracellular Vesicles of 2018; Théry et al., 2018), released from cells after fusion of multivesicular bodies with the plasma membrane, are composed of a lipid bilayer with surface molecules recognized by target cells, surrounding a heterogenous cargo which includes proteins, lipids, smaller metabolites and nucleic acids. Since this molecular cargo is protected from enzymatic degradation, sEVs are considered promising vehicles for the identification of potential biomarkers.

Placental sEVs (P‐sEVs) are of specific interest due to their potential to transmit signalling molecules to both maternal and foetal target tissues and to induce systemic effects (Mitchell et al., 2015; Tkach & Théry, 2016). Any changes in the placental microenvironment (e.g., availability of oxygen or nutrients) can influence their biogenesis, contents or bioactivity (Adam et al., 2017). Hence, P‐sEVs reflect foetoplacental metabolism (Miranda et al., 2018) in real‐time. The release of P‐sEVs into the maternal circulation begins in the first weeks of pregnancy (Tannetta et al., 2017), underscoring their potential as early‐pregnancy biomarkers. So far, the relevance of P‐sEV cargo (i.e., miRNA and proteins) has been actively investigated in the context of a variety of pregnancy‐related disorders such as preeclampsia (Tannetta et al., 2017), gestational diabetes (Herrera‐Van Oostdam et al., 2020; Salomon et al., 2016) and preterm birth (Broere‐Brown et al., 2020; Ermini et al., 2021; Mincheva‐Nilsson & Baranov, 2014; Landmann et al., 2006; Skotland et al., 2019). Despite the importance of lipids in foetal growth (Herrera & Ortega‐Senovilla, 2014) and their role as bioactive mediators in major cellular pathways and whole‐body metabolism (Bikman & Summers, 2011), no studies have systematically examined the lipidome of P‐sEVs across gestation, neither in normal pregnancies nor pregnancies affected by SGA.

One of the main objectives of the Human Placental Project (HPP) (https://www.nichd.nih.gov/research/supported/HPP/default) is to advance the innovation of real‐time methods for the monitoring of foetoplacental health across gestation. In the current study, we characterized the lipid composition of circulating placental sEVs throughout normal pregnancy, from first trimester to delivery. We further investigated whether the lipid content of placental sEVs differs in relation to birthweight and explored their utility for the identification of pregnancies with an SGA foetus. We focused on pregnant individuals with normotensive, term SGA pregnancies with no other major complications since they represent the largest population delivering an SGA infant. These women are at the highest risk of having FGR missed, due to the lack of routine ultrasound monitoring of foetal size in the 3rd trimester of pregnancy.

## 2 MATERIALS AND METHODS

### 2.1 Sample collection and clinical information

Normotensive pregnant individuals with (1) an appropriate‐for‐gestational age (AGA) or large‐for‐gestational age (LGA) foetus (*n* = 220) and (2) a small‐for‐gestational (SGA) foetus (*n* = 43) were provided by the Ontario Birth Study (OBS), an on‐going, prospective, longitudinal pregnancy cohort (www.ontariobirthstudy.com) at Sinai Health System, Toronto, Canada. Women with multiple gestations or pregnancies affected by chromosomal abnormalities, major malformations or smoking were excluded from the study. All participants provided written informed consent for the study. The study protocol was approved by the Mount Sinai Hospital (MSH)/Sinai Health System Research Ethics Board (REB number: 17‐0040‐E) observing the Declaration of Helsinki. Available information on maternal age, parity, gravidity, weight, height, highest blood pressure levels during pregnancy, gestational age at delivery and delivery mode were extracted by the OBS from the MSH patient records. Obesity was defined as maternal pre‐pregnancy body mass index ≥ 30 kg/m^2^. Similarly, data on infant sex, birthweight, birth length, head circumference, Apgar scores and umbilical artery pH at birth were collected from hospital records. Infant birthweight between the 10th and 90th percentile was defined as AGA, < 10th percentile as SGA, and > 90th percentile as LGA, using a Canadian standard population, standardized for sex and gestational age (Kramer et al., 2001). Within the SGA group, nine cases were < 3rd percentile, and as such considered severe SGA. Together AGA (92%) and LGA (8%) comprised the control group. Neonatal ponderal index was calculated as (birthweight in grams) × 100/(birth length in cm) (Gardosi et al., 2013). We obtained four blood samples from all individuals in the control group. Each participant from the SGA group provided 2–4 blood samples across pregnancy at 10–14 (G1, *n* = 41), 16–22 (G2, *n* = 26), and/or 26–32 (G3, *n* = 37) weeks’ gestation, and/or at delivery (G4, *n* = 36). All maternal blood samples were processed immediately after collection. Nine millilitres samples of blood were drawn into a vacutainer containing K2‐EDTA (Becton‐Dickson), inverted twice and retained on ice immediately after blood draw. Whole blood was separated into packed red cells, buffy coat and plasma within 30 min of collection by centrifuging at 3500 × *g* for 15 min at 4°C. Plasma was aliquoted into 1.5 mL screw cap vials, flash frozen in liquid N_2_ and stored at −80°C until sEV isolation. These collection and processing procedures were designed to minimize variations in plasma and sEV quality.

### 2.2 Small EV isolation

Small EVs were isolated from plasma by ultracentrifugation and filtration. Plasma (500 μL) was added to an equal volume of phosphate buffered saline (PBS) and centrifuged at 15,000 × *g* for 45 min at 4°C. Supernatant was transferred to ultracentrifuge tubes and centrifuged at 136,000 × *g* for 2 h at 4°C in a Sorvall MTX 150 Micro‐Ultracentrifuge (Thermo Scientific) using a S140 AT rotor (Thermo Scientific). Supernatant was removed and the pellet was resuspended in 500 μL PBS. Another 500 μL of PBS was added and suspension was filtered through a 0.22 μM filter (FroggaBio, Concord, Canada). The filtrate (total sEVs: T‐sEV) was aliquoted and stored at −80°C prior to use.

In pilot experiments using 27 samples from control (*n* = 25) and SGA pregnancies (*n* = 2), we compared the ultracentrifugation and filtration (UC+F) isolation method with a method that involves ultracentrifugation followed by discontinuous sucrose gradient centrifugation and filtration (UC+S+F). In the latter method adapted from Ermini et al. (2017), sEVs isolated from plasma by ultracentrifugation were layered on a sucrose gradient ranging from 0.25 to 2.5 M. After centrifugation at 110,000 × *g* for 16 h, fractions between 1 and 1.50 M sucrose were combined and spun at 91,000 × *g* for 2 h. Pellet was resuspended in PBS followed by filtration through a 0.22 μM filter. Placental (P)‐sEVs were then collected from filtrate by anti‐PLAP immunoprecipitation as described below. Subsequent NTA and lipidomic analyses of P‐sEVs isolated by either UC+F or UC+S+F did not reveal any significant differences in percentages of lipid classes examined (Figure S1). Hence, we selected the shorter UC+F method for the large‐scale isolation of sEVs from maternal plasma.

To isolate P‐sEVs, a 250 μL aliquot of total sEVs was incubated overnight on a rotator at 4°C with 2.5 μL of biotinylated anti‐human placental alkaline phosphatase (PLAP) monoclonal antibody (Invitrogen # MA512691catalogue number). Streptavidin agarose (50 μL; Novagen #69203) in PBS was added to each sample before rotating for 3 h at 4°C. Samples were then centrifuged at 12,000 × *g* for 1 min and supernatant was removed. Exosome Elution Buffer (300 μL Exoflow buffer‐2; System Biosciences) was added to the pellet before rotating for 3 h at room temperature, followed by centrifugation at 12,000 × *g* for 1 min and collection of the supernatant. This P‐sEV fraction was stored at −80⁰C prior to lipidomic mass spectrometry, immunoblotting and FACS analyses.

We also examined the impact of repeated freeze‐thawing of maternal plasma samples on sEV integrity, concentration, and lipid composition. Nanoparticle tracking analysis (NTA) demonstrated that maternal plasma samples frozen and thawed twice contained less P‐sEVs and more aggregates compared to P‐sEVs isolated from the same plasma samples immediately after collection, that is, one freeze‐thaw cycle (Figure S1b). Although no significant changes were observed in lipid class composition between P‐sEVs isolated from maternal plasma after one or two freeze‐thaw cycles, alterations were noted in specific lipid species such as Sphingomyelin (SM) (Figure S1c). Thus, only plasma that was frozen once (at collection) was used for all subsequent placental sEV isolations and lipidomic analyses.

### 2.3 Western blotting

Western blotting was employed to detect canonical sEV‐related proteins, including CD63, Alix and TSG101 (Mincheva‐Nilsson et al., 2016), as previously described (Ermini et al., 2021). Calnexin was used as a negative control while placental alkaline phosphatase indicated placental origin (Mincheva‐Nilsson & Baranov, 2014).

### 2.4 Flow cytometry

For flow cytometry, sEVs were conjugated to 4 μM aldehyde/sulphate latex beads (InVitrogen #A37304). Five μL of (4.1 g/100 mL) bead suspension was added to 100 μL of sEV suspension (8–10 × 10 [Figuera et al., 2008] sEVs per mL in PBS), mixed, and incubated for 15 min at room temperature (RT). Forty‐five microlitres of PBS was then added and the incubation continued for another 45 min. To stop the reaction, 100 μL of 2% (w/v) bovine serum albumin (BSA) in PBS was added. After 3 h of incubation, 0.5 mL of PBS was added and latex beads were pelleted at 2500 × *g* for 15 min. Latex beads were resuspended in 150 μL of 100 mM glycine in PBS and incubated for 30 min at RT. PBS was added (end volume 0.5 mL) and latex beads were collected by centrifugation at 2500 × *g* for 15 min at RT. Latex beads were resuspended in 100 μL of PBS for staining with combinations of 1:100 diluted PE‐conjugated mouse anti‐human CD63 (Novus #NBP2‐42225PE), APC‐conjugated mouse anti‐human PLAP (Novus #NB110‐3638APC) and AF488‐conjugated mouse anti‐human ApoB (Santa Cruz #sc13538) monoclonal antibodies for 30 min at 4°C. A Beckman Coulter Gallios flow cytometer was used for the analysis (minimal 10,000 events were collected). Unstained and single‐stained latex bound sEVs were used for gating. Accuracy of flow cytometry analysis was verified using the MIFlowCyt template (Table S1).

### 2.5 Transmission electron microscopy

Placental sEVs were suspended in an equal volume of 4% (v/v) paraformaldehyde. Sample preparation and imaging were conducted by the Nanoscale Biomedical Imaging Facility at the Hospital for Sick Children in Toronto, ON, Canada. Twenty microlitres of sample was deposited on clean, carbon‐coated TEM copper grids and incubated for 1 h. The grid was then fixed in 1% (v/v) glutaraldehyde for 5 min, washed in sterile distilled water and incubated in 2% (w/v) uranyl‐oxalate contrast solution at pH 7 for 10 min. After washing, the grid was embedded in 2% (w/v) methyl cellulose‐UA for 10 min on ice. Excess cellulose was removed, and samples were dried and imaged using an FEI Tecnai 20 transmission electron microscope (FEI Life Sciences‐Thermo Fisher Scientific).

### 2.6 Nanoparticle tracking analysis (NTA)

Measurements of sEV concentration and size were performed by NTA using a NanoSight NS300 (Malvern). For these analyses, placental sEV samples were 1:3 diluted in PBS. The measurements were performed with a 532 nm laser module at 22°C. Each sEV sample was vortexed for 3 s before being transferred to a 1 mL syringe. After priming the sample chamber with PBS, 150 μL of sample was loaded and three recordings of 30 s each were acquired at a camera level of 14. When necessary, flow rate was adjusted to have particles flow 5–10 s through the measurement window. After each recording, the sample chamber was flushed with 1 mL of PBS before loading the next sample. Recordings were analysed by NanoSight NTA version 3.4 software, using a detection threshold of 10 and all other settings set to automatic. Results were exported as csv files, giving the number of particles for each size in 1 nm increments, and read into R version 4.0.3 using the TidyNano library (https://journals.plos.org/plosone/article?id https://doi.org/10.1371/journal.pone.0218270). sEV number and size were calculated by taking the mean of the three recordings.

### 2.7 Lipid extraction

All lipidomic analyses, including lipid extraction and mass spectrometry, were conducted at the Analytical Facility for Bioactive Molecules (AFBM) at the Hospital for Sick Children, Toronto, ON, Canada. Analytical scientists of the AFBM were blinded to the study to avoid any subjective bias. The whole study was performed over 2 years with batches several months apart. One hundred microlitres of sEV suspension was diluted into 1 mL water, sonicated for 1 min and transferred to round bottom glass tubes. Lipid extraction was performed by adding 1 mL MeOH followed by 2 mL of CHCl_3_. Tubes were vortexed for 1 min, kept on ice for 10 min and then centrifuged for 10 min at 300 × *g*. The lower chloroform layer was then transferred to a fresh set of conical glass tubes. The remaining samples in the original round bottom tube were acidified with 40 μL of 0.1N HCl and subjected to another partitioning with CHCl_3_. The CHCl_3_ layer was recombined with the previous extract and resulting lipid extracts were dried under a stream of nitrogen. Residues were then reconstituted in 100 μL 10 mM ammonium acetate in 1:1 MeOH:DCM (dichloromethane) for mass spectrometry analysis.

### 2.8 Lipid mass spectrometry

Reconstituted lipid extract (50 μL) was automatically loaded and delivered to the electrospray ionization (ESI) source using a 1260 Infinity II LC System (Agilent Technologies) with a 50 μL sample loop. The mobile phase consisted of 10 mM ammonium acetate in MeOH:DCM. The total infusion time was 11 min per sample with the following flow rates: 0–7 min, 0.08 mL/min; 7–9 min, 0.1 mL/min; 9–11 min, 0.08 mL/min. The LC system was coupled to a SCIEX 6600‐TripleTOF mass spectrometer (SCIEX, Concord, Canada) equipped with a Turbo Ion Spray source. ESI was performed in the positive and negative mode. Typical ESI source parameters for both positive and negative modes were nebulizing gasses GS1 at 20, GS2 at 28, curtain gas at 30, ion spray voltage at +4500/−4500 V, declustering potential at +60/−80 V and temperature at 150°C. The atmospheric pressure chemical ionization probe was connected to a calibrant pump which delivers a mass calibration solution for MS and MS/MS. The assumption is made that all lipids within a class ionize and fragment with the same efficiency. The data‐independent MS/MS^All^ with SWATH acquisition was controlled by Analyst 1.7 software (SCIEX, Concord, Ontario). For both negative and positive mode, TOF‐MS involved 120 accumulation scans of 5.5 s per sample and mass range of 100–2000 m/z. The MS/MS^ALL^ collision energy (CE) for negative lipids was set to −10 V whereas for positive lipids, CE was set to 40 V. Lipid identification was carried out using LipidView and MarkerView Software—Version 1.3 beta and 1.3.1, respectively (SCIEX, Concord, Ontario). Identified lipids were cross‐checked against Lipid Maps and SwissLipid structural databases and only lipids present in both databases were included in the final analyses. Samples were analysed in batches of up to 40 samples with an instrument maintenance step at the end of each batch involving mass spectrometer ion source cleaning. The first and final two injections of each batch were bovine heart lipid extract QCs (Avanti Polar Lipids, Sigma, St Louis, US) to equilibrate and check the stability of the mass spectrometer during each run and to provide quality assurance between batches. Batches with a coefficient of variation of more than 50% based on QCs were reinjected. LipidView data were manually exported into Microsoft Excel. The Excel files were read into Python version 3.8/Pandas version 1.2.4. In pilot experiments using quantitative tandem mass spectrometry (Ermini et al., 2017), we found that the cholesterol amount in P‐sEVs remained constant with advancing gestation and did not alter in SGA. As it has been reported that sEVs are enriched in glycerophospholipids and sphingolipids (Skotland et al., 2019) we focused on these two lipid classes (Table S2). In addition to the base glycerophospholipid species, lyso‐, ether‐ (x O‐), and lyso‐ether‐ (Lx O‐) lipids were also captured. In cases where a sample had been injected multiple times, the injection with the highest mean peak intensity was chosen. Peak intensities were assumed to represent relative abundance of each lipid class and species. Finally, lipid abundance (lipid peak intensity) per million sEVs was calculated using the individual values for the number of sEVs per mL plasma.

### 2.9 Antibodies

Primary antibodies utilized include anti‐human PLAP (Abcam, SP‐15, ab16695, rabbit monoclonal [WB 1:1000]); biotinylated anti‐human PLAP (Thermo Fisher Scientific, 8B6.18, MA512691); anti‐humanCD63 (Santa Cruz Biotechnology, E‐12, sc‐365604, mouse monoclonal [WB 1:1000]); anti‐human ALIX (Thermo Fisher Scientific, PA5‐52873, rabbit polyclonal [WB 1:1000]); anti‐human TSG101 (Thermo Fisher Scientific, JJ0900, MA5‐32463, rabbit monoclonal [WB 1:5000]); anti‐human apolipoprotein B (R&D, AF3556, polyclonal goat antibody [WB 1:2000), and rabbit polyclonal anti‐human Calnexin (Novus Biologicals, Littleton, Colorado, USA, NB100‐1965 [WB 1:500]. Secondary antibodies were obtained from the Jackson Laboratory and included horseradish peroxidase (HRP)‐conjugated to goat anti‐rabbit IgG (111‐035‐144, [WB 1:3000]), and HRP‐conjugated to goat anti‐mouse IgG (115‐035‐146, [WB 1:3000]).

### 2.10 Data analysis

#### 2.10.1 Clinical data

Statistical analyses were performed using IBM SPSS Statistics Version 28.0. Categorical variables were analysed with the Chi‐square test, using exact *p‐*values. For the comparison of continuous variables in two groups, Student's *t* test or Mann‐Whitney *U* test was utilized, as appropriate. Data are presented as means (SD) or medians (IQR or range). *p*‐Values <  0.05 were considered statistically significant.

#### 2.10.2 NTA data

Since the NTA data failed the Shapiro‐Wilk test of normalcy, even after attempts at log‐transformation, non‐parametric tests were chosen. The unpaired two‐sample Wilcoxon test was used when comparing control versus SGA samples. The paired‐samples Wilcoxon test was employed when comparing different gestational timepoints. Benjamin‐Hochberg correction was used to account for multiple comparisons.

#### 2.10.3 Lipid data

All statistical analyses of lipidomic data were performed using the R version of Metaboanalyst (R 3.6.3, RStudio 1.3.1073, Windows 10 × 86 64‐w64‐mingw32/x64 [64‐bit], MetaboAnalystR 3.0). Data were log‐transformed and normalized using “quantile normalization.” In an unsupervised approach, principal component analysis (PCA) was used to explain the variation in the data using the prcomp package (version 3.6.2). In a supervised approach, partial least square discriminant analysis (PLS‐DA) was carried out. The PLS regression was performed using the plsr function, which is included in the R pls package (version 2.8‐1). Classification and cross‐validation were carried out using the caret package's equivalent wrapper function. A false discovery rate (FDR) of less than 0.05 was utilized for Student's *t*‐test while the fold change (cut‐off = 2) was estimated by using the univariate analysis of variance (ANOVA). For heatmaps, the top 25 significantly altered species between SGA and control samples based on ANOVA/*t*‐test were used. The random forest technique, implemented in the MetaboAnalystR package (version 3.0), was used to produce multivariate receiver operating characteristic (ROC) curves and AUCs utilizing random sub‐sampling cross‐validation, in which 70% of the data were utilized for model training, and the remaining 30% (holdout) for model testing.

## 3 RESULTS

### 3.1 Maternal characteristics and perinatal outcomes

Maternal characteristics and perinatal outcomes of control participants (*n* = 195) with normal pregnancies versus pregnant women with an SGA foetus (*n* = 41) are shown in Table 1. Maternal age and maternal pre‐pregnancy body mass index (BMI) levels were similar in both groups. Pregnant women with an SGA foetus were more often primiparous. Most participants in both the control and SGA groups identified themselves as being of White European (59% vs. 44%, respectively) or Ashkenazi Jewish (19% vs. 18%, respectively) origin. In both groups, approximately 9% of individuals were East Asian. The only statistically significant difference in ethnic group distribution between the study groups was a higher prevalence of Southeast Asian ethnic origin in the SGA (15%) group versus controls (3%). Pre‐pregnancy weight was lower in mothers with an SGA foetus, but there were no differences with respect to maternal height and pre‐pregnancy BMI. Although both groups remained normotensive throughout pregnancy, systolic and diastolic blood pressure levels were significantly higher among the women with an SGA foetus. As expected, neonatal ponderal indices and head circumferences were lower in the SGA group. The birth weight (g) of new‐born infants was 2611 with a mean (SD, 257) in the SGA group and 3464 (SD, 391) in the control (AGA/LGA) group. The median (IQR) relative birth weight (percentile) was 5.6 (3.2/7.2) in the SGA group and 51.2 (30.9/72.1) in the control group. There were no statistically significant differences between control and SGA groups with respect to gestational age at delivery or the frequencies of different delivery modes. Umbilical artery pH was similar in both groups. While not significant due to the SGA sample size, 5% of SGA infants had 5 min Apgar scores < 7, in contrast to < 0.5% of control infants (*p* = 0.13).

**TABLE 1:** Maternal and perinatal characteristics of participants.

Maternal or perinatal characteristic	Control	SGA	*p‐*Value
	*n* = 195	*n* = 41	
Maternal age, years	33 (22–45)	33 (24–43)	0.614
Primiparous (%)	96 (46.4)	31 (75.6)	0.003
Maternal ethnicity (%)	[160]	[34]	0.049
White European	95 (59.4)	15 (44.1)	0.128
Jewish	31 (19.4)	6 (17.6)	0.818
East Asian	15 (9.4)	3 (8.8)	1.000
Latin American/Hispanic	5 (3.1)	3 (8.8)	0.147
Southeast Asian	4 (2.5)	5 (14.7)	0.009
West Asian	3 (1.9)	1 (2.9)	0.540
Maternal pre‐pregnancy weight, kg	63.3 (58.5/69.9) [189]	57.6 (55.3/65.3) [38]	0.017
Maternal height, cm	167.6 (161.3/169.5) [87]	167.0 (157.0/168.0) [19]	0.216
Body mass index, kg/m^2^	23.4 (21.5/25.7) [189]	23.2 (20.9/25.1) [38]	0.462
Systolic blood pressure, mmHg	120.0 (114.0/129.5)	124.0 (120.0/131.0)	0.051
Diastolic blood pressure, mmHg	76.0 (70.0/80.0)	80.0 (73.0/86.0)	0.022
Delivery mode (%)			
Spontaneous vaginal delivery	148 (75.9)	30 (73.2)	0.842
Planned caesarean section	27 (13.8)	5 (12.2)	0.812
Unplanned caesarean section	20 (10.3)	6 (14.6)	0.585
Gestational age at birth, weeks	39.6 (38.8/40.4)	39.4 (38.4/40.3)	0.120
Preterm birth < 37 weeks’ gestation	3 (1.5%)	1 (2.4%)	0.536
Infant sex, % male/female	105 (53.8)/90 (46.2)	19 (46.3.5)/ 22 (53.7)	1.000
Birth weight (BW), g	3464.5 (± 391.1)	2611 (± 257)	<0.001
Birth weight percentile	51.2 (30.9/72.1)	5.6 (3.2/7.2)	<0.001
Severe SGA (BW < 3rd centile)	0	9 (21%)	
LGA (birth weight > 90th centile)	18 (8.2%)	0	
Birth length, cm	51.0 (50.0/53.0) [161]	49.0 (47.5/50.0) [32]	<0.001
Ponderal index, g/cm^3^	2.56 (2.40/2.73) [161]	2.23 (2.14/2.41) [32]	<0.001
Head circumference, cm	35.0 (34.0/36.0) [133]	33.0 (33.0/34.0) [29]	<0.001
Umbilical artery pH	7.21 (7.17/7.25) [173]	7.21 (7.13/7.28) [40]	0.622
5 min Apgar < 7	2 (0.9) [215]	2 (4.7) [43]	0.145

*Note*: Values for continuous variables are median (range or IQR), mean (SD) or percentages (%). Number of subjects is shown in square brackets if different.

Abbreviation: SGA, small for gestational age.

### 3.2 Validation of placental sEV isolation

**FIGURE 1:**
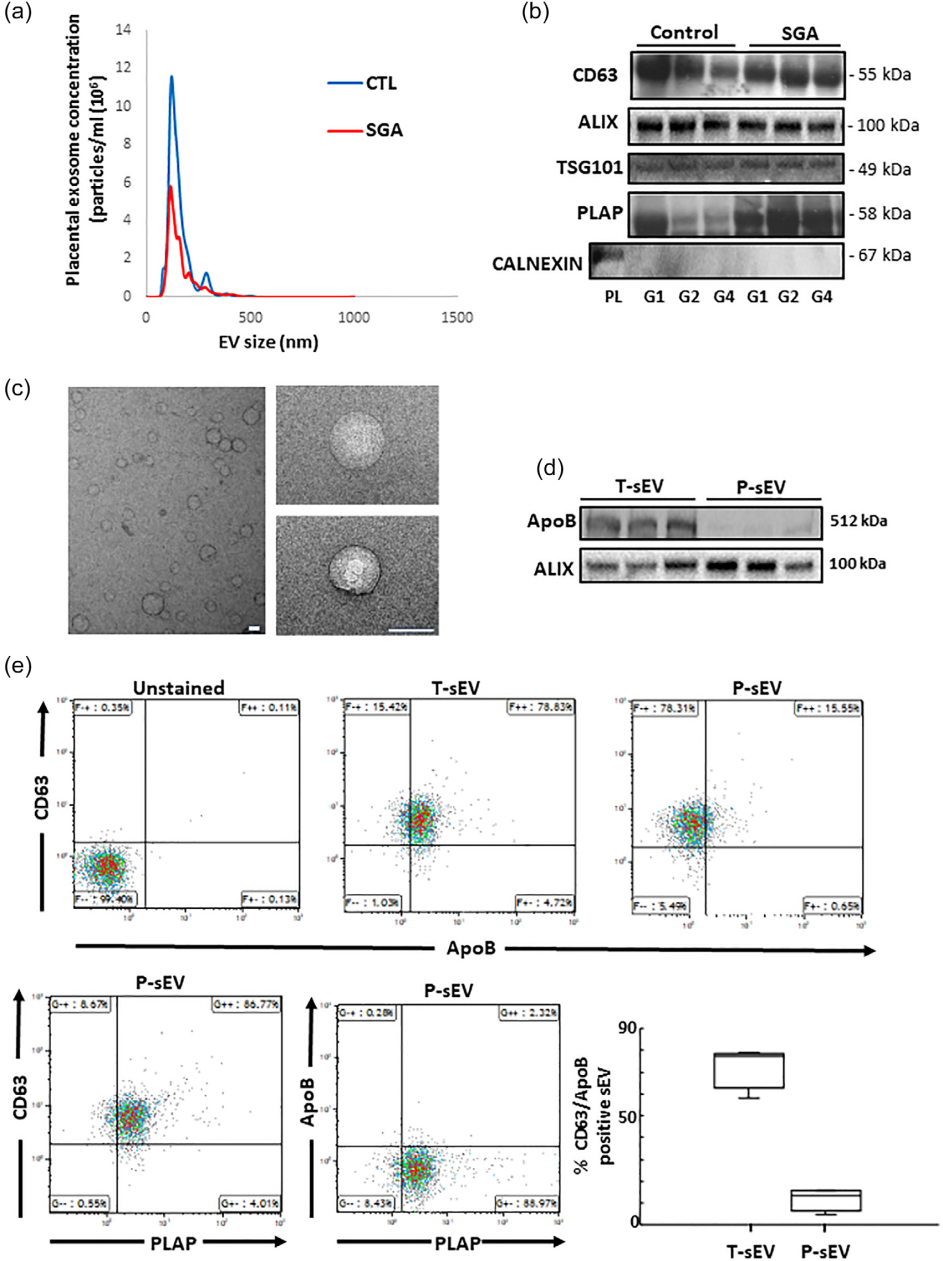
Characterization and validation of placental sEVs isolated from maternal plasma. (a) Representative NTA measurements of P‐sEVs isolated from maternal plasma of normal and SGA pregnancies. (b) Immunoblots for CD63, ALIX, TSG101, PLAP and CALNEXIN (negative control) of P‐sEVs isolated from maternal plasma of normal and SGA pregnancies at gestational windows G1, G2, G3 and G4 of pregnancy (*n* = 3 different sEV isolations). (c) Representative electron micrographs of P‐sEVs isolated from maternal plasma of normal pregnancy. Bar = 100 nm. (d) Immunoblots for ApoB and ALIX of total (T‐sEV) and placental (P‐sEV) extracellular vesicles isolated from maternal plasma of normal pregnancy (*n* = 3 different sEV isolations). (e‐upper panels) Representative flow cytometry density plots for CD63 and ApoB in T‐sEVs and P‐sEVs isolated from maternal plasma of normal pregnancy. (e‐lower panel, left) Representative cytometry density plots for CD63, PLAP and ApoB in P‐sEVs from maternal plasma of normal pregnancy. (e‐lower panel, right) Percentage of double positive CD63/ApoB EVs in total and placental sEVs (*n* = 4 different sEV isolations). EV, extracellular vesicles; NTA, nanoparticle tracking analysis; sEV, small EV; SGA, small‐for‐gestational age.

After establishing optimal procedures for small EV isolation from maternal plasma of 25 controls and two SGA pregnancies, we isolated T‐sEVs and P‐sEVs from maternal plasma samples collected across 195 control and 41 SGA pregnancies. Figure 1 shows a representative NTA of freshly isolated placental sEV samples. In general, NTA of sEVs displayed a bell‐shaped, right‐skewed size distribution with the mode at 130.4 nm (95% confidence interval [CI]: 129.1–131.8 nm), the median at 152.2 nm (95% CI: 150.9–153.4 nm) and an interquartile range of 122.8 to 196.5 nm (95% CI: 121.9–123.7 nm to 194.5–198.4 nm), conforming to guidelines on sEV size (Théry et al., 2018). The quality of isolated sEVs was confirmed in every fifth G1 to G4 plasma sample from the same pregnant mother by immunoblotting for CD63, ALIX, TSG101 (all three enriched in sEVs), PLAP (to identify sEVs of placental origin) and calnexin (absent in most sEVs and used as negative control) (Figure 1b). TEM analysis confirmed the typical spherical shape of isolated sEVs (Figure 1c). Furthermore, to ensure that isolated sEVs are devoid of lipoprotein contamination (e.g., HDLs, LDLs, VDLs, IDLs, ranging in size from 5 to 1000 nm), we examined Apolipoprotein B content (ApoB, involved in forming the outer shell structure of lipoproteins) in both isolated T‐sEV and P‐sEV preparations. Western blotting and flow cytometry showed the presence of small amounts of ApoB in T‐sEVs, but a complete absence in P‐sEVs (Figure 1d,e). Triacylglycerols and cholesterol esters, major constituents of lipid droplets and lipoproteins (Skotland et al., 2019), were rarely detected in P‐sEVs (data not shown).

### 3.3 SGA pregnancies differ by having larger, but fewer placental sEVs in the maternal circulation

**FIGURE 2:**
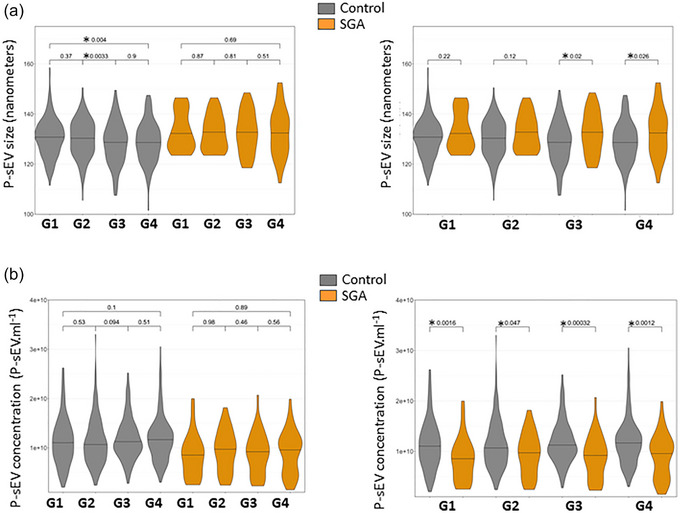
Size and concentration of circulating placental sEVs across normal and SGA pregnancies. Violin plots of placental extracellular vesicle size (a) and concentration (b) in maternal plasma of normal and SGA pregnancies at gestational windows G1, G2, G3 and G4 of pregnancy measured with NTA. Control *n* = 195 and SGA *n* = 41. NTA, nanoparticle tracking analysis; sEV, small EV; SGA, small‐for‐gestational age. One‐way ANOVA and Tukey posthoc test, * *p* < 0.05.

NTA was performed on each individual P‐sEV sample from each gestational age timepoint in the control and SGA pregnancies. This was the basis for assessing P‐sEV plasma concentration and size distribution across gestational ages. NTA data were filtered to include only particles between 50 and 175 nm in size, which comprised on average, 85.2% (95% CI: 84.8%–85.6%) of all < 200 nm sEV particles (Théry et al., 2018). In control maternal plasma there was a trend towards smaller P‐sEVs with advancing gestation with statistically significant differences between G2 and G3 as well as G1 and G4 (Figure 2a, left panel). In contrast, no changes in P‐sEV size were found in the maternal plasma of the SGA group at any gestational timepoint (Figure 2a, left panel). When control and SGA pregnancies were compared, there was a trend towards larger sized P‐sEVs circulating in the plasma of the SGA pregnancy group relative to control pregnancies at all gestational timepoints, with statistically significant changes relative to control at G3 and G4 (Figure 2a, right panel). While the number of P‐sEVs circulating in maternal blood did not change with gestational age in either control or SGA pregnancies (Figure 2b, left panel), SGA pregnancies had fewer P‐sEVs circulating in the maternal blood compared to control pregnancies at all 4 time points (Figure 2b, right panel). This was already evident in the first trimester of pregnancy (G1), with the difference persisting until delivery (Figure 2b, right panel). Overall, circulating P‐sEVs in SGA pregnancies were fewer in number throughout gestation but larger in size in late pregnancy.

### 3.4 Lipid composition of circulating placental sEVs changes with advancing gestation

In total, we identified 948 phospholipid species by shotgun MS/MS^All^. The most abundant phospholipids in circulating P‐sEVs from normal and SGA pregnancies were PC (∼61%–65%), followed by sphingomyelin (SM; ∼23%–24%), phosphatidylinositol (PI; ∼3%–5%), phosphatidylserine (PS; ∼3%–5%), phosphatidic acid (PA; ∼2%–3%), phosphatidylglycerol (PG; ∼0.5%), phosphatidylethanolamine (PE; ∼0.5%) and ceramide (Cer; ∼2%) (Table 2). Ether‐ and lyso‐phospholipids were relatively minor lipid constituents of circulating P‐sEVs (Table S1). Lysophosphatidylcholine (LPC) and lysophosphatidic acid (LPA) constituted 1%—3% of their lipid class. In contrast, lysophosphatidylinositol (LPI) made up 15%—21% of total PI. Ether‐linked LPI, PA, PI, and PS constituted approximately 15%, 38%, 45% and 55% of their lipid class, respectively, while PE and PC ethers were hardly detectable or absent. For the major phospholipids, PC and SM, species containing one unsaturated fatty acid chain were most abundant. PC16:0/18:1 (∼15%), PC16:0/18:2 (∼28%), PC 18:0/18:2 (∼10%) were the dominant PC species while SMd18:1/16:0, SMd18:1/20:0 and SMd18:1/22.0 constituted approximately 6%, 30% and 13% of total SM, respectively. SMd18:1/24:1 and SMd18:1/24:2 (∼6% each) were the dominating very‐long‐chain SM species. No major differences in P‐sEV lipid class distribution were observed across gestation. Only PE content in P‐sEVs was statically greater in G3 and G4 compared to G1 controls. PI and PS abundance was significantly increased when comparing control and SGA pregnancy samples at G1 to G4 timepoints.

**TABLE 2:** Phospholipid composition of placental small EVs circulating in the bloodstream of pregnant women at different gestational ages.

	Ceramide	SM	PC	PE	PG	PI	PS	PA
Control G1	1.71 ± 0.001	24.18 ± 0.16	64.21 ± 0.51	0.43 ± 0.01	0.43 ± 0.01	3.19 ± 0.22	3.31 ± 0.18	2.50 ± 0.22
Control G2	1.77 ± 0.001	24.19 ± 0.15	64.72 ± 0.45	0.41 ± 0.01	0.42 ± 0.02	3.14 ± 0.24	3.01 ± 0.16	2.30 ± 0.20
Control G3	1.77 ± 0.002	23.97 ± 0.18	65.08 ± 0.42	0.54 ± 0.0^b^	0.40 ± 0.02	2.95 ± 0.18	3.12 ± 0.18	2.14 ± 0.17
Control G4	1.77 ± 0.001	23.64 ± 0.14	65.28 ± 0.44	0.63 ± 0.0^b^	0.42 ± 0.03	2.93 ± 0.19	3.11 ± 0.21	2.21 ± 0.17
SGA G1	1.77 ± 0.003	23.05 ± 0.69	60.77 ± 2.05	0.53 ± 0.05	0.67 ± 0.13	4.65 ± 1.05	5.44 ± 0.8^a^	3.11 ± 0.69
SGA G2	1.76 ± 0.001	22.86 ± 0.30	62.08 ± 1.01	0.52 ± 0.0^a^	0.62 ± 0.0^a^	4.06 ± 0.3^a^	5.22 ± 0.4^a^	2.87 ± 0.41
SGA G3	1.76 ± 0.001	22.78 ± 0.29	62.70 ± 1.08	0.59 ± 0.02	0.58 ± 0.0^a^	3.86 ± 0.4^a^	4.95 ± 0.5^a^	2.74 ± 0.38
SGA G4	1.76 ± 0.001	22.51 ± 0.30	62.90 ± 1.01	0.63 ± 0.02	0.58 ± 0.0^a^	3.90 ± 0.3^a^	4.97 ± 0.5^a^	2.78 ± 0.35

*Note*: Percent of total lipids. Data are means ± SEM, *n* = 198 control and 41 SGA samples at each gestational (G1–G4) window.

Abbreviations: EV, extracellular vesicles; PA, phosphatidic acid; PC, phosphatidylcholine; PE, phosphatidylethanolamine; PG, phosphatidylglycerol; PI, phosphatidylinositol; PS, phosphatidylserine; SM, sphingomyelin.

^a^ *p* < 0.05 SGA versus control of respective gestational window.

^b^ *p* < 0.05 G3‐4 versus G1; unpaired *t*‐test with Welch correction.

**FIGURE 3:**
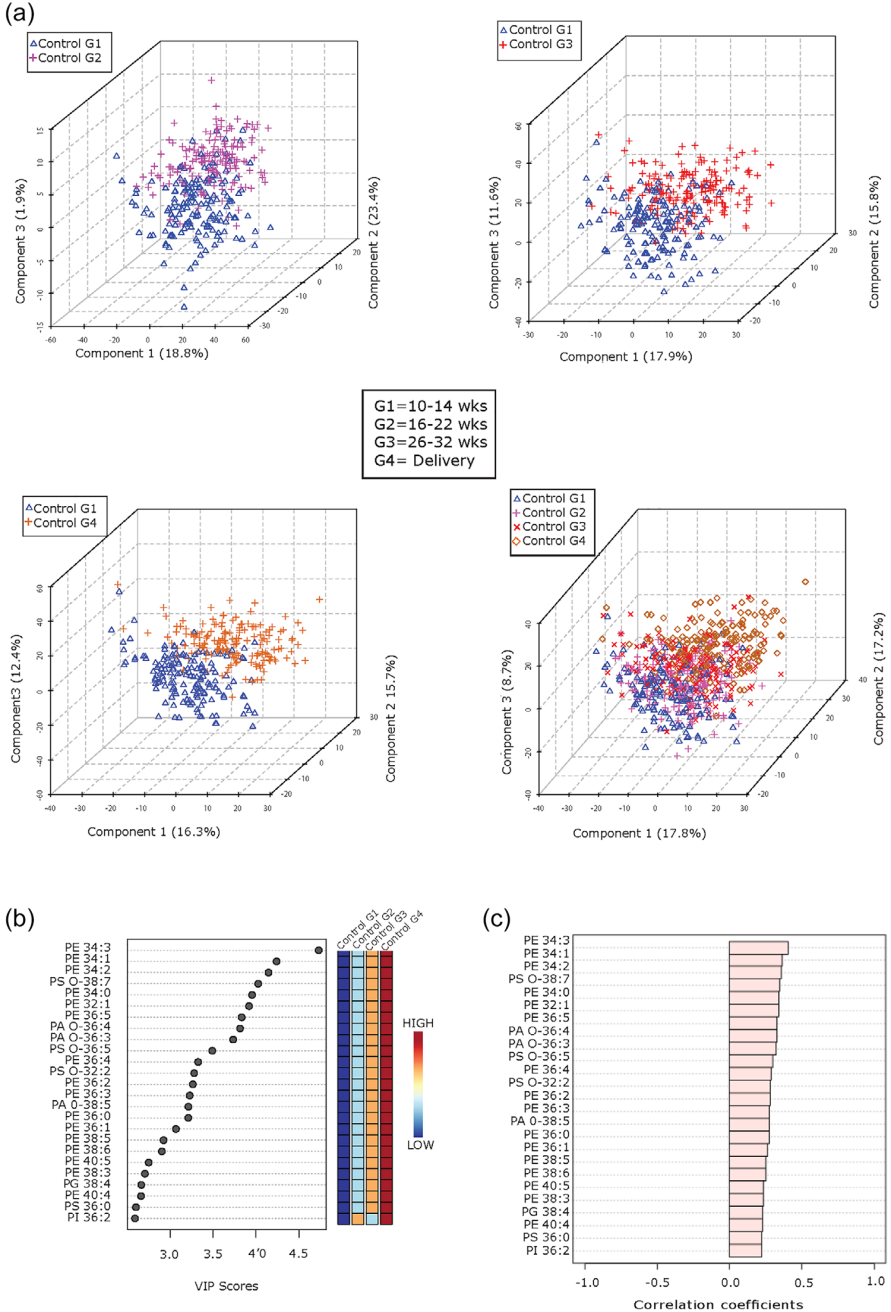
Lipid composition of circulating placental sEVs changes with gestation in normal pregnancy. (a) Partial least squares‐discriminant analysis (PLS‐DA) plots of global lipid profiles of P‐sEVs between gestational time points G1 versus G2, G3 and G4, respectively; and combined G1, G2, G3 and G4. (b) Top 25 lipids in P‐sEVs identified by PLS‐DA that exhibit changes across pregnancy (G1 to G4). Coloured boxes on the right indicate the relative concentrations of the corresponding lipid in each gestational group. Importance of each lipid change is represented by their variable importance in projection (VIP) score. (c) Pearson (r) correlation patterns of the identified top 25 lipids in circulating P‐sEVs across pregnancy (G1 to G4). PLS‐DA, partial least square discriminant analysis; P‐sEVs, placental sEVs; sEV, small EV.

Next, we interrogated the global phospholipid species profiles of P‐sEVs isolated from maternal plasma of normal pregnancies across various gestational time points (G1–G4). Partial least‐squares discriminant analysis (PLS‐DA) demonstrated distinct lipid signatures of P‐sEVs at different gestational windows as shown by the differential clustering of samples collected at G1 versus G2, G3 and G4 in 3D plots (Figure 3a). The greatest separation of clusters was observed between G1 and G4, indicating major differences in P‐sEV lipid composition (Figure 3a, bottom panels). To identify the most prominent lipidomic changes in P‐sEVs circulating in maternal plasma across normal pregnancy, variable interdependent parameters (VIP) scores, which reflect a variable's importance within the PLS‐DA model, were calculated for each P‐sEV lipid species that displayed variation depending on the different gestational time points (i.e., a higher score reflects a larger contribution to the model and to the separation). The 25 highest VIP scores for lipid species changes (Figure 3b) and 25 most significant lipid species that increase linearly across normal pregnancy were identified (Figure 3c). Several PE species and a few ether‐linked PA and PS species were found to increase in circulating P‐sEVs with advancing gestation (Figure 3b).

### 3.5 Maternal characteristics affect lipid composition of circulating placental sEVs

**FIGURE 4:**
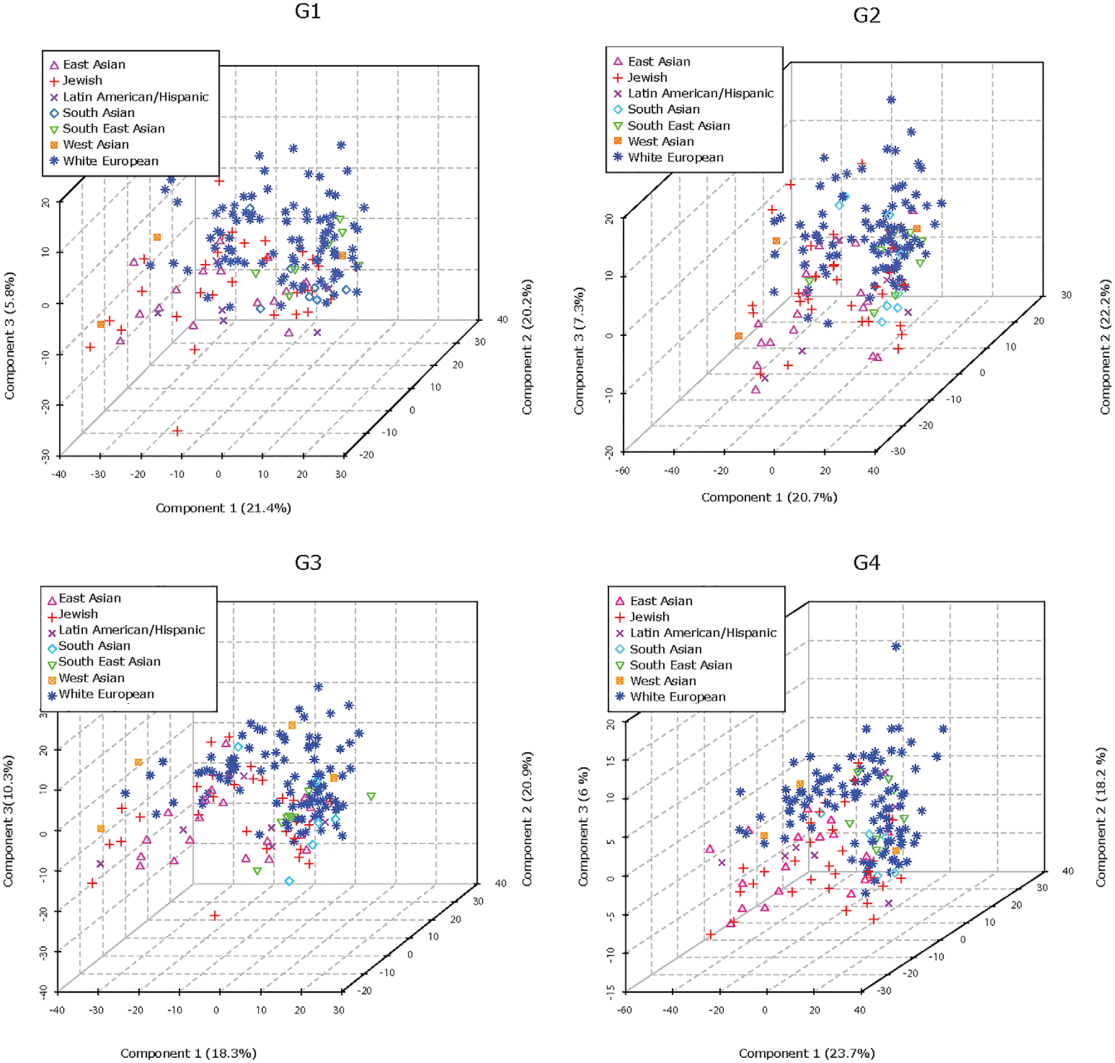
Maternal ethnicity affects lipid composition of circulating placental sEVs across normal pregnancy. PLS‐DA plots separate global lipid profiles of P‐sEVs isolated from plasma of normal pregnancies based on ethnic background at gestational timepoints G1, G2, G3 and G4 of pregnancy. PLS‐DA, partial least square discriminant analysis; P‐sEVs, placental sEVs; sEV, small EV.

**FIGURE 5:**
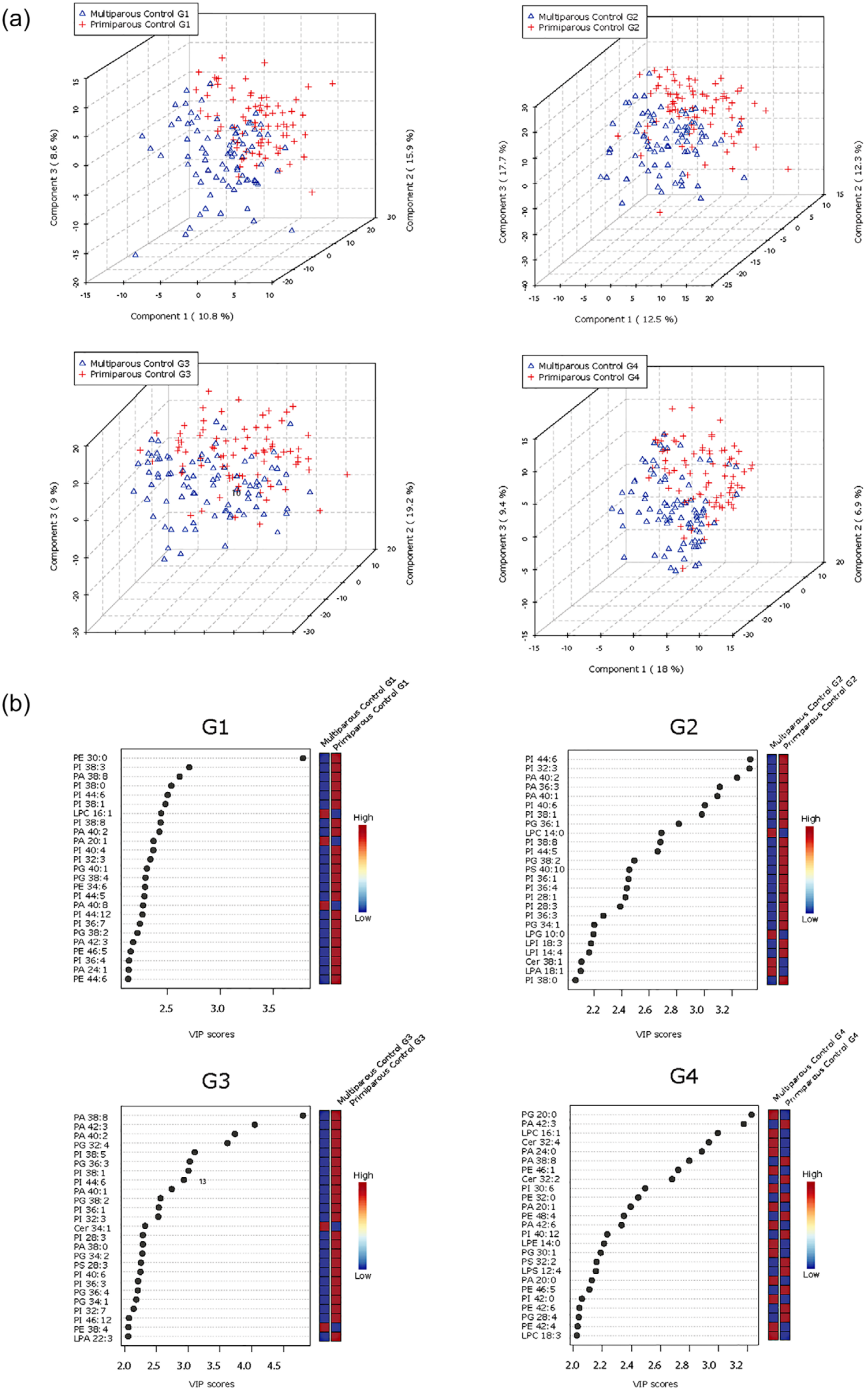
Parity‐based changes in lipid composition of circulating placental sEVs across normal pregnancy. (a) PLS‐DA plots separate global lipid profiles of P‐sEVs isolated from plasma of normal pregnancies based on parity (primi‐ vs. multipara) at gestational time points G1, G2, G3 and G4 of pregnancy. (b) Top 25 lipids identified by PLS‐DA that differ between primi‐ and multiparous controls across gestation (G1 to G4). Colored boxes on the right indicate the relative concentrations of the corresponding lipid in each parity group. Importance of each lipid difference is represented by their variable importance in projection (VIP) score. PLS‐DA, partial least square discriminant analysis; P‐sEVs, placental sEVs; sEV, small EV.

Maternal ethnicity is known to influence offspring size, and the large sample size (*n* = 195) in the control cohort permitted the inclusion of maternal self‐reported ethnicity as a variable. PLS‐DA analysis revealed that samples from mothers with White European and East Asian background, and to a lesser extent those with South Asian or Southeast Asian background, were separable at all gestational time points (G1–G4), suggesting ethnicity‐associated variations in the lipidome of maternal circulating P‐sEVs throughout normal pregnancies (Figure 4). Maternal BMI and parity are also known to affect foetal size. Hence, we examined the associations of these risk factors with P‐sEV lipid profiles across gestation in control pregnancies. Interestingly, we found no differences in P‐sEV lipid signatures between obese mothers with a BMI ≥ 30 kg/m^2^ as compared to mothers with a BMI < 30 kg/m^2^ (data not shown). However, distinct clustering of P‐sEV global lipid profiles of primiparous versus multiparous mothers was observed across gestation (Figure 5a **)**. VIP scores analysis showed increases in specific PI, PA, PE and PG species in P‐sEVs from normal pregnancies at G1, G2 and G3 windows in primiparous individuals as compared to multiparous as identified by associated heatmaps (Figure 5b). At G4, the differences in P‐sEV lipid profiles between primi‐ and multiparous participants exhibited a different pattern, with distinct PA, PE and lysophospholipid species being either increased or decreased in primiparous versus multiparous women (Figure 5b). Of note, P‐sEVs from multiparous women had higher concentrations of Cer 38:1, 34:1 and 32:4 at G2, G3 and G4, respectively, whereas primiparous women had higher Cer 32:2 concentrations in their P‐sEVs at G4.

### 3.6 Lipid composition of circulating placental sEVs and foetal sex

Like maternal characteristics and risk factors, foetal sex is an important pregnancy‐related variable with an established impact on foetal growth in utero, birthweight and other perinatal outcomes (Broere‐Brown et al., 2020). Interestingly, in our study population, no statistically significant differences in P‐sEV lipids composition were observed in control (Figure S2a) pregnancies between female versus male foetus at any gestational timepoint.

### 3.7 Lipid composition of circulating placental sEVs depends on infant birthweight

**FIGURE 6:**
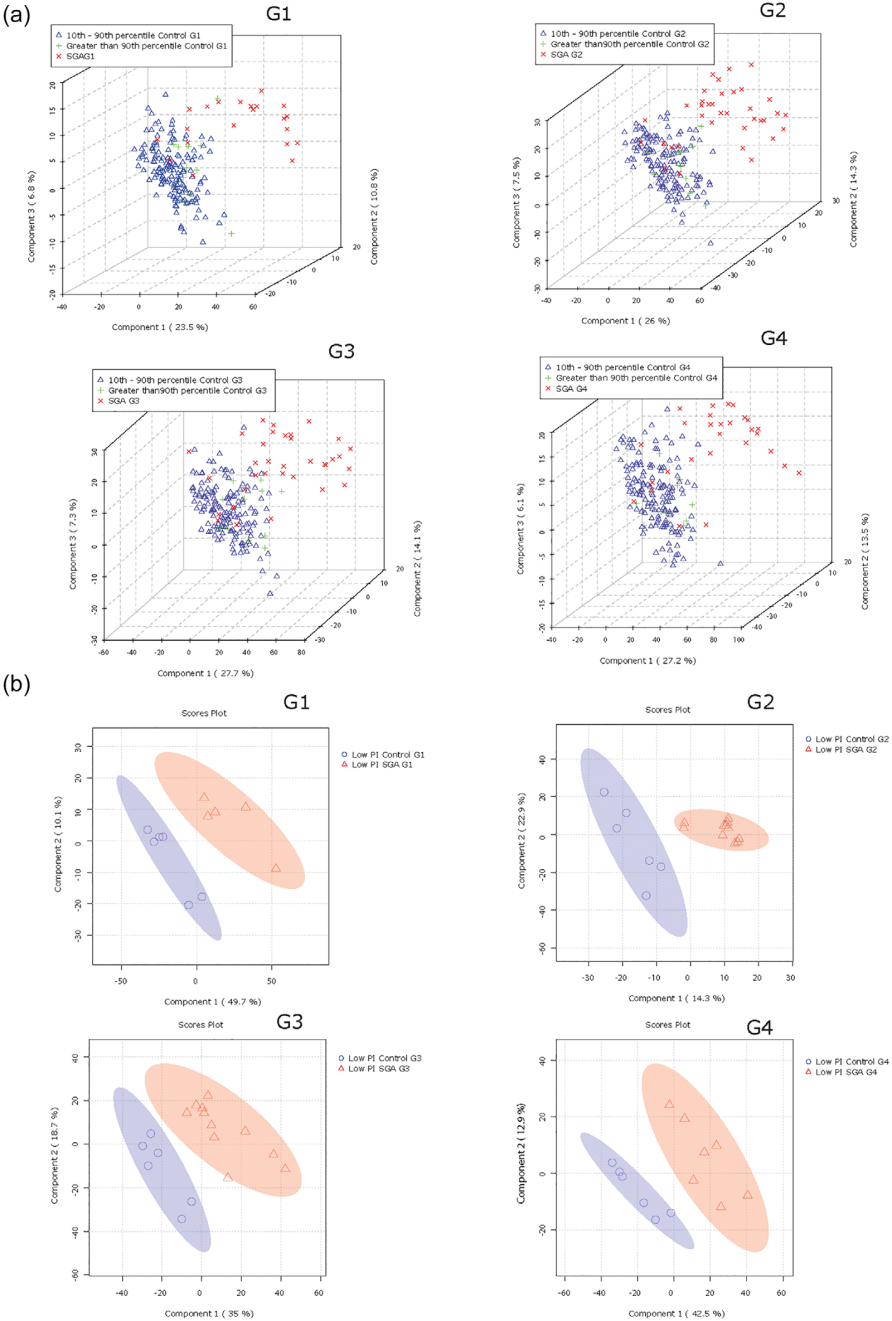
Lipid composition of circulating placental sEVs differs between normal and small‐for‐gestational age (SGA) pregnancies. All P‐sEVs isolated from maternal blood were categorized into three groups according to infant birthweight percentile: Small‐for‐gestational age (SGA; < 10th percentile), appropriate‐for‐gestational age (AGA; 10th–90th percentile) and large‐for‐gestational age (LGA; > 90th percentile). (a) PLS‐DA plots separates global lipid profiles of P‐sEVs between AGA versus. LGA versus SGA samples at different gestational timepoints (G1 to G4) of pregnancy. (b) Sparse PLS‐DA plots showing the separation according to placental sEV lipids of control versus SGA samples at different gestational timepoints (G1 to G4) of pregnancy according to low (< 2.2 g/m^3^) neonatal ponderal index (PI). PLS‐DA, partial least square discriminant analysis; P‐sEVs, placental sEVs; sEV, small EV.

Next, we examined the relationships between offspring birthweight and lipid composition of P‐sEVs in the maternal circulation. PLS‐DA analysis demonstrated that the global lipid profile of circulating P‐sEVs from the SGA pregnancy group separated from the control samples already at G1, and even more pronounced at G2, with similar separation persisting also at G3 and G4 (Figure 6a). In addition, to explore whether any differences could be seen in the lipid composition of P‐sEV in pregnancies in relation to differing foetal growth patterns, we also examined the AGA and LGA control pregnancies separately. Lipid profiles of circulating P‐sEVs from control mothers with an LGA foetus appeared to separate, albeit to a much lesser extent, as indicated by the clustering of these samples between the SGA and AGA groups (Figure 6a). Considering that primiparity is a significant risk factor of SGA, we analysed the differences in sEV lipid profiles separately in the primipara and multipara, and observed a separation in SGA lipid profile relative to controls in both primi‐ and multiparous participants (Figure S3). Likewise, we examined changes in sEVs lipid profiles separately in the White Caucasian participants and found a distinct separation in lipid profiles between SGA and control groups (Figure S4). We continued by categorizing our study participants according to the offspring ponderal index at birth, defined as the ratio of birthweight to birth length cubed (Landmann et al., 2006). The ponderal index is an anthropometric parameter that aids in the differentiation between symmetric and asymmetric growth restriction (Villar et al., 1990). In line with our findings related to birthweight percentiles, global lipid profiles of circulating P‐sEVs at each gestational timepoint (G1–G4) differed when comparing normal and SGA pregnancies with an infant ponderal index < 2.2 g/m^3^ (Figure 6b). Together, these observations support the hypothesis that lower birthweight and foetal thinness characterizing SGA pregnancies, associate with distinct lipid signatures of circulating P‐sEVs. Similar to control pregnancies, in SGA pregnancies, some differences in a variety of placental sEV lipid species were observed between male versus female foetuses across gestation, but statistical significance for these differences was lost after correcting for multiple comparisons (Figure S2b).

**FIGURE 7:**
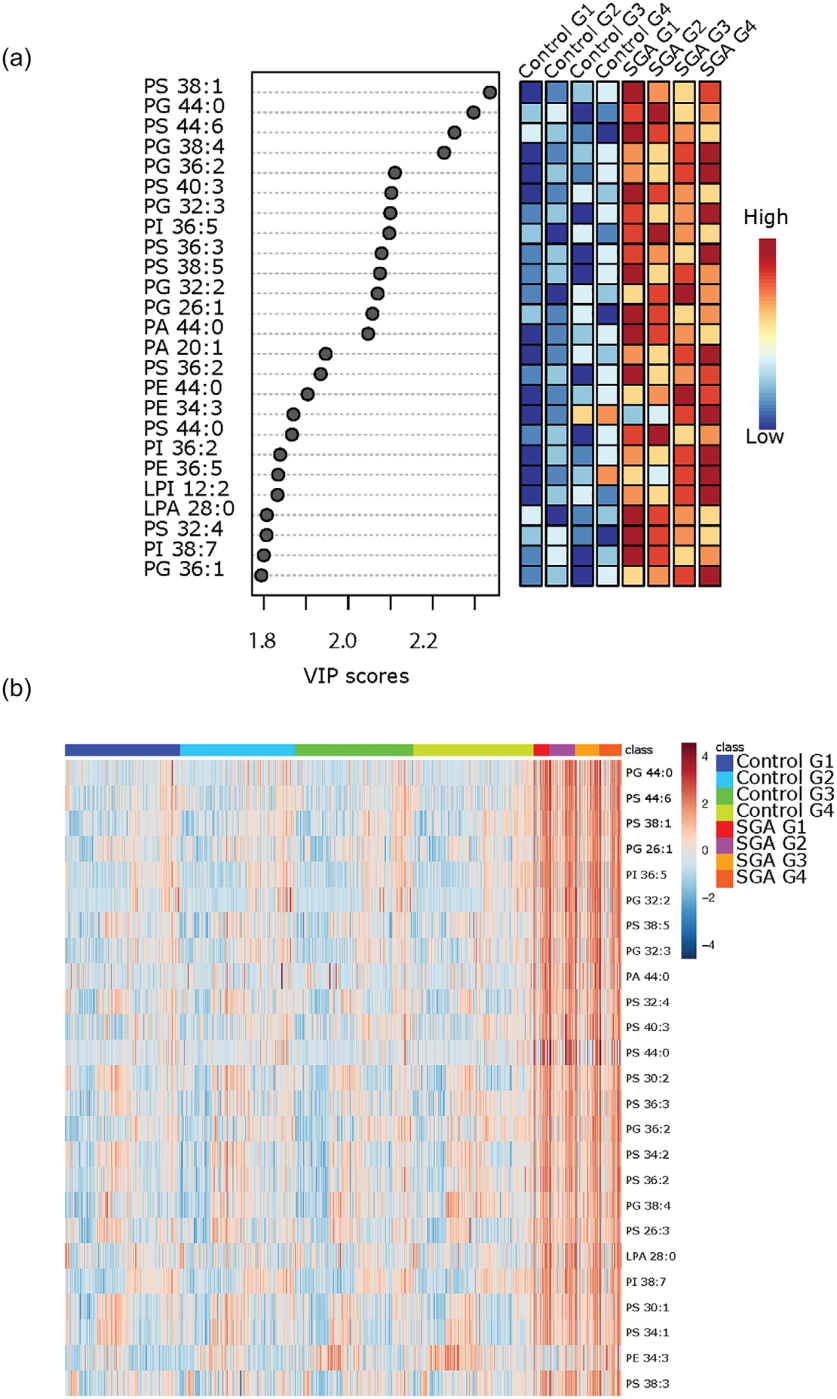
Most significant variances in lipid composition of circulating placental sEVs among normal and small‐for‐gestational age (SGA) pregnancies. (a) Top 25 lipids identified by PLS‐DA that differ between control (normal) versus SGA pregnancies across gestation. Colored boxes on the right indicate the relative concentrations of the corresponding lipid in each group. Importance of each lipid difference is represented by their variable importance in projection (VIP) score. (b) Heatmap showing the top 25 lipids that differ between placental sEVs isolated from maternal plasma of control (normal) versus SGA pregnancies at gestational timepoints G1 to G4. PLS‐DA, partial least square discriminant analysis; sEV, small EV

PLS‐DA analysis followed by VIP scores revealed that, except for PE 34:3, placental sEVs from SGA pregnancies had significantly higher levels of distinct PS and PG species as well as a few PA and PI species at all gestational timepoints (Figure 7a). Heatmap analysis confirmed similar top 25 lipid changes between the control and SGA pregnancy groups, with PS species being particularly abundant in P‐sEVs from SGA pregnancies across pregnancy (Figure 7b).

### 3.8 Lipid profile of placental sEVs in the maternal circulation predicts the birth of an SGA infant

**FIGURE 8:**
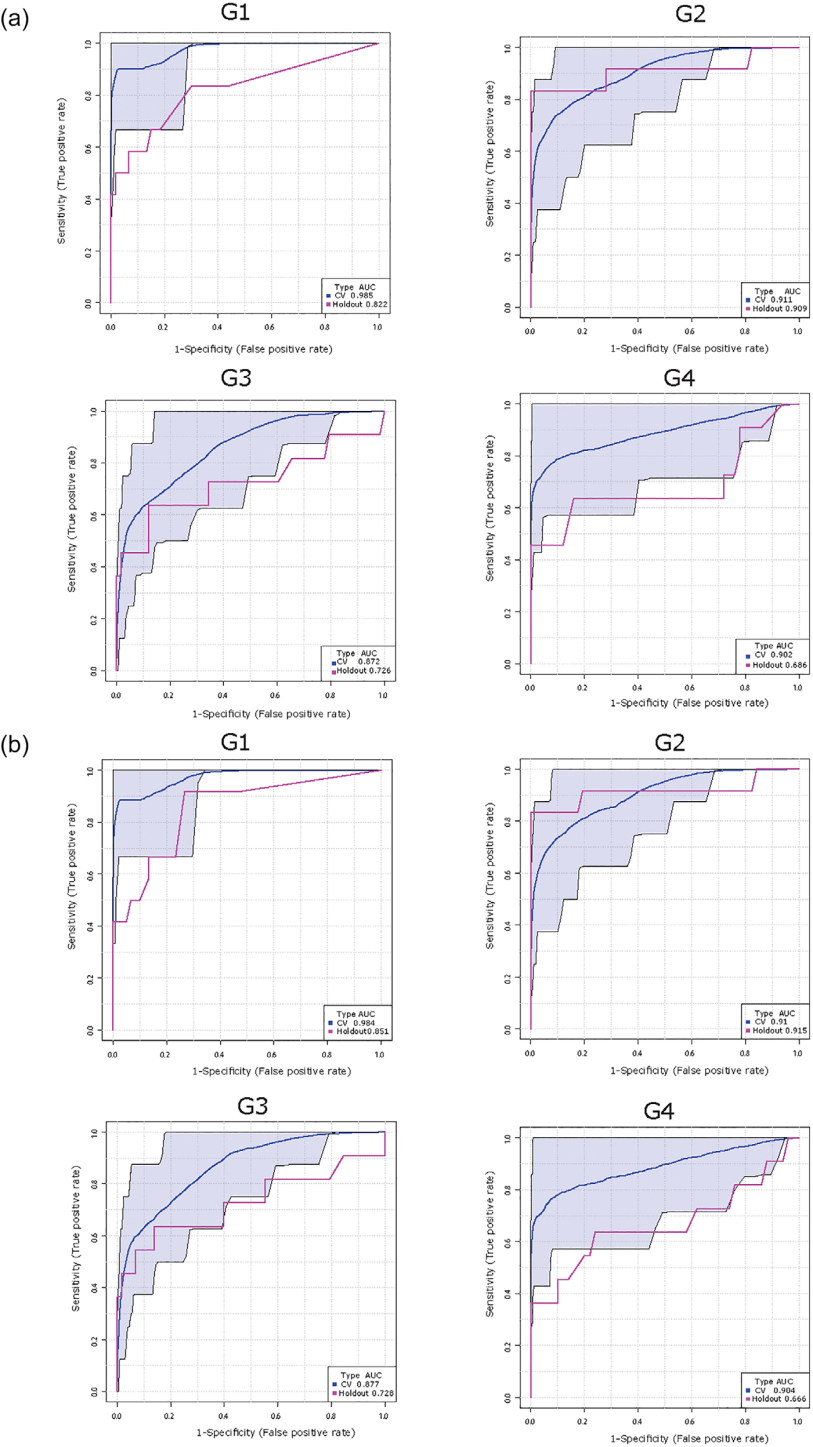
Lipid composition of circulating placental sEVs predicts the birth of a small‐for‐gestational age (SGA) infant. Receiver operating characteristic curves for the Random Forest classifier and area under the curve (AUC) scores for SGA prediction. Top 25 differentially expressed placental sEV lipids in control (normal) versus SGA pregnancies with and without maternal clinical (maternal age, BMI, and parity) characteristics were used for model development. (a) Prediction of SGA using top 25 differentially expressed P‐sEV lipids at different gestational timepoints (G1 to G4) without maternal clinical data. (b) Prediction of SGA using top 25 P‐sEV lipids at different gestational timepoints (G1 to G4) combined with maternal clinical variables. BMI, body mass index; P‐sEVs, placental sEVs; sEV, small EV.

To examine the diagnostic capacity of identified lipid signatures in P‐sEVs, we constructed ROC curves for cross validation and holdout experiments for the random forest prediction models trained by combining the top 25 P‐sEV lipids varying between control and SGA pregnancies (identified in Figures 7a,b) at different gestational timepoints. Random forest prediction models of these 25 distinct P‐sEV lipids were performed with and without basic maternal characteristics (i.e., maternal age, BMI and parity). The AUC values demonstrate that the best predictive power was achieved at G1 and G2 (holdout AUC of 0.822 and 0.909, respectively) without inclusion of maternal variables (Figure 8a), and holdout AUC 0.851 or 0.915 at G1 and G2 with inclusion of maternal risk factors (Figure 8b). The predictive accuracy of maternal characteristics alone, without the inclusion of P‐sEV lipids, was poor throughout pregnancy (Figure S5).

## 4 DISCUSSION

In the current study, we demonstrate changes in lipid composition of placenta‐derived sEVs circulating in the maternal plasma of normal pregnancies across gestation, from first trimester to delivery, and according to maternal characteristics such as parity and ethnicity. We also show that, when compared to control pregnancies with an AGA/LGA foetus, SGA pregnancies are characterized by less placental sEVs in the maternal circulation throughout pregnancy, and, importantly, a distinctly different lipid composition of the sEVs. Our multivariate prediction model suggests that a unique lipid signature of placenta‐derived sEVs in the maternal circulation, in combination with their concentration and known maternal risk factors, can be used to predict SGA diagnosis early in pregnancy.

A major strength of our study is the longitudinal sampling of maternal plasma via the OBS at four different time points across pregnancy, and access to detailed characterization of subjects including maternal and new‐born clinical outcomes. Moreover, considering the recognized challenges in sEVs research (Théry et al., 2018), we have undergone a rigorous method development phase to ensure isolation of high‐purity placenta‐derived sEVs, including the demonstration of absence of lipid droplets and/or lipoprotein contaminants that could confound our lipid analyses (Skotland et al., 2017). Our data on sEV quality in relation to plasma sample storage suggest that researchers should be cautious over the use of samples that have been subjected to multiple freeze‐thaw cycles when aiming at accurate analyses of sEV cargo.

To our knowledge, only one previous study has analysed placental sEVs in pregnancies with an SGA foetus (Miranda et al., 2018). Although this study did not examine the sEV cargo, it demonstrated that the contribution of placental sEVs to the total number of sEVs circulating in maternal and umbilical cord plasma at term was significantly reduced in pregnancies with a foetal birthweight < 3rd centile and abnormal foetoplacental Doppler indices when compared to AGA or SGA pregnancies with foetal birthweight between 3rd and < 10th centiles (Miranda et al., 2018). These observations are in line with our NTA findings; however, we observed a reduced number of placental sEVs in the maternal circulation of all SGA samples examined and not only in severe SGA cases as previously reported (Miranda et al., 2018). When SGA and severe SGA sEV groups were separated, the significant reduction in sEV number in the SGA group persisted, while in the severe SGA there was a trend towards decrease; which may be explained by the limited number of severe SGA cases used in this study (Figure S6). NTA was performed on all control (*n* = 195) and SGA (*n* = 41) samples enabling more accurate assessment of the quantity of placenta‐derived sEVs circulating in the maternal plasma of all tested groups. It is quite plausible that a lower placental mass, typical of SGA pregnancies, may contribute to the reduced number of placental sEVs released into the maternal circulation. Decreased levels of placental ceramide and sphingosine‐1‐phosphate (S1P), which characterize FGR placentae (Chauvin et al., 2015), could also play a role, as ceramide is known to promote sEV biogenesis (Verderio et al., 2018) and S1P has been reported to stimulate the maturation of sEV‐multivesicular endosomes (Kajimoto et al., 2013).

Although the relevance of lipids as key metabolic molecules that contribute to the progression of a normal pregnancy is established (Fakhr et al., 2021; Herrera & Ortega‐Senovilla, 2014; Nagamatsu et al., 2014), to our knowledge, this is the first study reporting gestational changes in the lipidome of sEVs of specifically placental origin circulating in maternal blood. To date, only a few studies have characterized the lipid composition of sEVs in biological fluids like urine (Skotland et al., 2017) and seminal fluid (Brouwers et al., 2013). Herein, we show that PC and SM are the most abundant phospholipids in circulating placental sEVs isolated from both normal and SGA pregnancies, with PS, PI, PA, PE and Cer being smaller lipid constituents. Our ex vivo data agree with the phospholipid composition of sEVs secreted by primary isolated trophoblast cells showing PC abundance (∼56%) with SM (∼12%), PI (∼10%), PS (∼5 %) and PE (∼12%) being present in lower amounts (Ouyang et al., 2016). PG, PA, LPA and LPC were minor components. A similar phospholipid composition was reported for sEVs isolated from placental explants’ culture supernatants with SM, PC, PI, and PS being the most abundant (Baig et al., 2013). Since sEVs originate as intraluminal vesicles (late endosomes) of multivesicular bodies, their lipid composition can be assumed similar to that of intraluminal vesicles (Skotland et al., 2017), which is primarily composed of CHOL, SM and PC (Skotland et al., 2019). The presence of PC, SM and PS in circulating placental sEVs is consistent with PC, SM and PS being major lipids in placental tissue (Brown et al., 2016; Huang et al., 2013) and with SM being enriched in lipid rafts of syncytiotrophoblast surface membranes that function as a platform for intraluminal vesicle formation (Ermini et al., 2017).

From the MS/MS^ALL^ shotgun lipidomic analyses, PE and ether‐linked PS and PA species in circulating placental sEVs emerged as key changes occurring towards the end of gestation in normal pregnancies. PE is abundant in the placenta (Brown et al., 2016; Huang et al., 2013) and its presence, although minor, in placenta‐derived sEVs (Baig et al., 2013; Ouyang et al., 2016) is an anticipated finding, specifically considering the placenta being a highly metabolic organ that secretes extracellular vesicles throughout pregnancy (Adam et al., 2017). To the best of our knowledge, gestational changes in placental PE content have not been investigated or reported. Ether‐linked lipids have been less studied, but they have been identified in sEVs (Skotland et al., 2019). PC and PE ethers are thought to promote fusion of multivesicular bodies with the cell membrane, thereby stimulating sEV release (Phuyal et al., 2015). Also, they are bioactive signalling molecules that engage in crosstalk with sphingolipids and cholesterol and have been implicated in cardiovascular, (Graessler et al., 2009), cancer (Chen et al., 2016) and metabolic disease (Schooneveldt et al., 2022). Mass spectrometry has enabled discovery of atypical ether‐linked species in PA, PS and PI but their biological functions are unknown (Ivanova et al., 2010). It is plausible that changes in PS and PA ethers observed in late versus early pregnancy in circulating placenta‐derived sEVs during gestation reflect placental metabolic adaptations required for advancing pregnancy.

In our study population, several maternal characteristics were found to be associated with variations in lipid composition of circulating placental sEVs throughout pregnancy. Typically, perinatal complications associated with placental dysfunction such as stillbirth, preeclampsia and foetal growth restriction are more common in nulliparous than in parous women. Although the precise mechanisms behind this phenomenon remain elusive, enhanced maternal immunological and cardiovascular adaptation, leading to improved placentation and foetal growth in multiparous women, are considered likely factors (Goldman‐Wohl et al., 2019), and these could contribute to the parity‐associated differences seen in the placental sEV lipid cargo during normal pregnancies. In particular, differences in placental sEV ceramide species between primi‐ and multiparous women are an intriguing finding, considering that sphingolipids are centrally involved in placental development (Eliyahu et al., 2007; Singh et al., 2012), and ceramide metabolism is severely disturbed in preeclamptic and FGR placentae (Ausman et al., 2018; Bailey et al., 2017; Chauvin et al., 2015; Ermini et al., 2021; Melland‐Smith et al., 2015). Interestingly, SGA and control pregnancies were characterized by differential lipid profiles in both primiparous and multiparous women, making it unlikely that the birth‐size‐associated differences are solely due to confounding by parity.

Maternal ethnicity has been reported to affect foetal size, with South (Stanfield et al., 2012) and East Asian (Sletner et al., 2015) mothers having lighter and thinner babies as compared to White European mothers. Our data revealed clear variations in circulating placental sEV lipid profiles between women with different ethnic backgrounds, for example, East and South Asian and Jewish versus White European mothers, who comprise a significant portion of OBS participants. While no information is available about foetoplacental lipidomic differences due to ethnicity, variations in maternal metabolome (Sovio et al., 2015) and in maternal lipid and lipoprotein metabolism (Koukkou et al., 1994) have been reported between ethnic groups (Lindsay et al., 2015). Unfortunately, it was not possible to use relative birth weight adjusted for ethnicity in the present study. Hence, it is plausible that the somewhat higher prevalence of Southeast Asian mothers in the SGA group may have influenced our findings. Further studies utilizing other cohorts with different ethnic composition are warranted to confirm our results.

Although studies have reported that maternal obesity is associated with changes in placental lipid metabolism (Calabuig‐Navarro et al., 2017), placental sEV excretion (Elfeky et al., 2017), and maternal and cord plasma lipid profiles (Patel et al., 2018), we were surprised to find no differences in circulating placental sEV lipid content and composition in obese versus non‐obese control pregnancies. This could be due to the relatively low frequency of obesity in our study population from which women with obesity‐related pregnancy complications, such as hypertensive disorders and diabetes, had been excluded. Similarly, it was unexpected that no statistically significant differences were found with respect to foetal sex. The existence of such differences cannot be ruled out, however, considering that sEV lipid profile patterns appeared somewhat different when comparing male versus female pregnancies within the control and SGA groups.

Appropriate lipid signalling is vital for proper placental development (Eliyahu et al., 2007; Nagamatsu et al., 2014). Moreover, maternal fat accrual in early pregnancy and the development of physiological hyperlipidemia in late pregnancy, ensure the availability of lipids to the growing foetus (Herrera & Ortega‐Senovilla, 2014). Not surprisingly, metabolomic studies have reported alterations in both maternal (Miranda et al., 2018) and foetal (Miranda et al., 2018) plasma lipid metabolites in SGA pregnancies with and without clinical signs of FGR, with the degree of metabolic disruption increasing with the severity of SGA subtype (Miranda et al., 2018). Although it is widely accepted that a spectrum of derangements occurring in early placentation are central in SGA pregnancy, little is known about the role of placental lipid metabolism in foetal growth. We have previously demonstrated disrupted sphingolipid metabolism in FGR placentae, leading to decreased placental levels of ceramide and sphingosine‐1‐phosphate (S1P)—potent signalling lipids that balance cell growth and death (Chauvin et al., 2015). Our present data in SGA pregnancies reveal persistent alterations in circulating placental sEV lipid composition, spanning from early gestation to term, suggesting a potential placental metabolic component in SGA pathogenesis. We found that several glycerophospholipid species including PS and PG were particularly enriched in SGA placental sEVs throughout pregnancy, a finding consistent with a recent metabolomics study showing heightened levels of glycerophospholipids in maternal plasma collected at 20 weeks’ gestation in SGA pregnancies (Morillon et al., 2021). PS molecules are known to function as an “eat me” signal for macrophages (Segawa & Nagata, 2015) on the surface of apoptotic bodies and microvesicles, and also mediate microvesicle internalization into target tissues (Wei et al., 2016). Hence, if PS molecules enriched in placental sEVs of SGA pregnancies are located on the outer lipid leaflet, or later “flipped” to the sEV surface, it is tempting to speculate that enhanced clearance of these sEVs by macrophages (Skotland et al., 2017), or via tissue uptake, could partially explain the reduced sEV number in the maternal circulation of SGA pregnancies. Despite our earlier data on alterations in placental sphingolipid metabolism in SGA pregnancies (Chauvin et al., 2015), in the present study, sphingolipid changes did not dominate in the sEV lipid profile changes related to SGA. Differences in gestational age at birth might contribute to this discrepancy, as most cases in our previous study on placental tissue lipidomics in FGR pregnancies were pre‐term, while in the present study on placental sEV we analysed term SGA cases.

The requirement for sensitive and specific screening methods for FGR is urgent. As the current available intervention is early delivery, misdiagnosing healthy but constitutionally small foetuses as growth restricted, and as such, exposing them to the potential harms of unnecessary interventions and preterm birth, poses a substantial threat. Interestingly, a systematic review of 15 metabolomics studies suggested that lipid metabolites should be validated up to the second trimester for the early prediction of SGA in different settings (Leite et al., 2019). Although our large‐scale shotgun lipidomic study identified several differential lipid metabolites, it is unlikely that the sole assessment of placental sEV lipid cargo could determine the final diagnosis of SGA or FGR. However, our results from random forest prediction models using the differential lipids are promising with respect to early identification of pregnancies at the highest risk of poor foetal growth. Our ROC curves suggest high sensitivity and specificity for the prediction of SGA via assessment of placental exosome lipid signatures and the concentration of placenta‐derived exosomes in the maternal circulation, in early (10–14 weeks) pregnancy.

In conclusion, our study provides the first evidence of changes in circulating placental sEV lipid content in normal pregnancies according to gestational age, parity and maternal ethnic background. Of potential clinical relevance, we identified unique lipid signatures of circulating placental sEVs in SGA pregnancies that can be detected in the first trimester of pregnancy, in addition to SGA‐associated changes in the overall number and size of circulating EVs across gestation. Analysis of placental exosome lipid profile appears to be a promising novel means to improve the early identification of pregnancies that could benefit from more intensive growth monitoring throughout pregnancy. Our results call for the development of analytical tools that would allow for rapid quantification of placental sEV concentration in maternal plasma and their selected lipid cargo. Testing of sEV lipid cargo profiles in sufficiently powered clinical studies with ethnically diverse study populations and systematic inclusion of comprehensive data on maternal and foetal risk factors (i.e., fetoplacental Doppler parameters, foetal growth velocity, placental weight and neonatal anthropometric/metabolic parameters), would move such predictive testing forward into clinical use.

## AUTHOR CONTRIBUTIONS

**Miira Klemetti**: Data curation; formal analysis; investigation; methodology; visualization; writing—original draft. **Ante Pettersson**: Data curation; formal analysis; investigation; methodology; validation; visualization. **Aafaque Khan**: Data curation; formal analysis; methodology; software; visualization; writing—review and editing. **Leonardo Ermini**: Investigation; methodology; supervision; validation. **Tyler Porter**: Investigation; methodology; validation. **Michael Litvack**: Validation; visualization. **Sruthi Alahari**: Visualization; writing—review and editing. **Stacy Zamudio**: Conceptualization; writing—review and editing. **Nicholas Illsley**: Conceptualization; writing—review and editing. **Hannes Röst**: Data curation; formal analysis; supervision; validation; writing—review and editing. **Martin Post**: Conceptualization; data curation; funding acquisition; methodology; project administration; resources; supervision; visualization; writing—review and editing. **Isabella Caniggia**: Conceptualization; data curation; formal analysis; funding acquisition; methodology; project administration; resources; software; supervision; visualization; writing—original draft; writing—review and editing.

## CONFLICT OF INTEREST STATEMENT

All authors declare no competing interests.

## Supporting information

Supporting Information

Supporting Information

Supporting Information

## Data Availability

The data that support the findings of this study are available from the corresponding author upon reasonable request. The data that supports the findings of this study are available in the supplementary material of this article.

## References

[jev212413-bib-0001] Adam, S. , Elfeky, O. , Kinhal, V. , Dutta, S. , Lai, A. , Jayabalan, N. , Nuzhat, Z. , Palma, C. , Rice, G. E. , & Salomon, C. (2017). Review: Fetal‐maternal communication via extracellular vesicles—Implications for complications of pregnancies. Placenta, 54, 83–88. 10.1016/j.placenta.2016.12.001 27939894

[jev212413-bib-0002] Arcangeli, T. , Thilaganathan, B. , Hooper, R. , Khan, K. S. , & Bhide, A. (2012). Neurodevelopmental delay in small babies at term: A systematic review. Ultrasound in Obstetrics & Gynecology : The Official Journal of the International Society of Ultrasound in Obstetrics and Gynecology, 40(3), 267–275. 10.1002/uog.11112 22302630

[jev212413-bib-0003] Ausman, J. , Abbade, J. , Ermini, L. , Farrell, A. , Tagliaferro, A. , Post, M. , & Caniggia, I. (2018). Ceramide‐induced BOK promotes mitochondrial fission in preeclampsia. Cell Death & Disease, 9(3), 298. 10.1038/s41419-018-0360-0 29463805 PMC5833856

[jev212413-bib-0004] Bahado‐Singh, R. O. , Yilmaz, A. , Bisgin, H. , Turkoglu, O. , Kumar, P. , Sherman, E. , Mrazik, A. , Odibo, A. , & Graham, S. F. (2019). Artificial intelligence and the analysis of multi‐platform metabolomics data for the detection of intrauterine growth restriction. PLoS One, 14(4), e0214121. 10.1371/journal.pone.0214121 30998683 PMC6472728

[jev212413-bib-0005] Baig, S. , Lim, J. Y. , Fernandis, A. Z. , Wenk, M. R. , Kale, A. , Su, L. L. , Biswas, A. , Vasoo, S. , Shui, G. , & Choolani, M. (2013). Lipidomic analysis of human placental syncytiotrophoblast microvesicles in adverse pregnancy outcomes. Placenta, 34(5), 436–442. 10.1016/j.placenta.2013.02.004 23465879

[jev212413-bib-0006] Bailey, L. J. , Alahari, S. , Tagliaferro, A. , Post, M. , & Caniggia, I. (2017). Augmented trophoblast cell death in preeclampsia can proceed via ceramide‐mediated necroptosis. Cell Death & Disease, 8(2), e2590. 10.1038/cddis.2016.483 28151467 PMC5386461

[jev212413-bib-0007] Bikman, B. T. , & Summers, S. A. (2011). Ceramides as modulators of cellular and whole‐body metabolism. The Journal of Clinical Investigation, 121(11), 4222–4230. 10.1172/JCI57144 22045572 PMC3204836

[jev212413-bib-0008] Broere‐Brown, Z. A. , Adank, M. C. , Benschop, L. , Tielemans, M. , Muka, T. , Gonçalves, R. , Bramer, W. M. , Schoufour, J. D. , Voortman, T. , Steegers, E. A. P. , Franco, O. H. , & Schalekamp‐Timmermans, S. (2020). Fetal sex and maternal pregnancy outcomes: A systematic review and meta‐analysis. Biology of Sex Differences, 11(1), 26. 10.1186/s13293-020-00299-3 32393396 PMC7216628

[jev212413-bib-0009] Brouwers, J. F. , Aalberts, M. , Jansen, J. W. , van Niel, G. , Wauben, M. H. , Stout, T. A. , Helms, J. B. , & Stoorvogel, W. (2013). Distinct lipid compositions of two types of human prostasomes. Proteomics, 13(10‐11), 1660–1666. 10.1002/pmic.201200348 23404715

[jev212413-bib-0010] Brown, S. H. , Eather, S. R. , Freeman, D. J. , Meyer, B. J. , & Mitchell, T. W. (2016). A lipidomic analysis of placenta in preeclampsia: Evidence for lipid storage. PLoS One, 11(9), e0163972. 10.1371/journal.pone.0163972 27685997 PMC5042456

[jev212413-bib-0011] Calabuig‐Navarro, V. , Haghiac, M. , Minium, J. , Glazebrook, P. , Ranasinghe, G. C. , Hoppel, C. , Hauguel de‐Mouzon, S. , Catalano, P. , & O'Tierney‐Ginn, P. (2017). Effect of maternal obesity on placental lipid metabolism. Endocrinology, 158(8), 2543–2555. 10.1210/en.2017-00152 28541534 PMC5551552

[jev212413-bib-0012] Chauhan, S. P. , Rice, M. M. , Grobman, W. A. , Bailit, J. , Reddy, U. M. , Wapner, R. J. , Varner, M. W. , Thorp, J. M., Jr , Leveno, K. J. , Caritis, S. N. , Prasad, M. , Tita, A. T. N. , Saade, G. , Sorokin, Y. , Rouse, D. J. , & Tolosa, J. E. , & MSCE, for the Eunice Kennedy Shriver National Institute of Child Health and Human Development (NICHD) Maternal‐Fetal Medicine Units (MFMU) Network . (2017). Neonatal morbidity of small‐ and large‐for‐gestational‐age neonates born at term in uncomplicated pregnancies. Obstetrics and Gynecology, 130(3), 511–519. 10.1097/AOG.0000000000002199 28796674 PMC5578445

[jev212413-bib-0013] Chauvin, S. , Yinon, Y. , Xu, J. , Ermini, L. , Sallais, J. , Tagliaferro, A. , Todros, T. , Post, M. , & Caniggia, I. (2015). Aberrant TGFβ signalling contributes to dysregulation of sphingolipid metabolism in intrauterine growth restriction. The Journal of Clinical Endocrinology and Metabolism, 100(7), E986–E996. 10.1210/jc.2015-1288 25942476

[jev212413-bib-0014] Chen, X. , Chen, H. , Dai, M. , Ai, J. , Li, Y. , Mahon, B. , Dai, S. , & Deng, Y. (2016). Plasma lipidomics profiling identified lipid biomarkers in distinguishing early‐stage breast cancer from benign lesions. Oncotarget, 7(24), 36622–36631. 10.18632/oncotarget.9124 27153558 PMC5095026

[jev212413-bib-0015] Ciobanu, A. , Rouvali, A. , Syngelaki, A. , Akolekar, R. , & Nicolaides, K. H. (2019). Prediction of small for gestational age neonates: Screening by maternal factors, fetal biometry, and biomarkers at 35–37 weeks' gestation. American Journal of Obstetrics and Gynecology, 220(5), 486.e1–486.e11. 10.1016/j.ajog.2019.01.227 30707967

[jev212413-bib-0016] Conde‐Agudelo, A. , Papageorghiou, A. T. , Kennedy, S. H. , & Villar, J. (2013). Novel biomarkers for predicting intrauterine growth restriction: A systematic review and meta‐analysis. BJOG : An International Journal of Obstetrics and Gynaecology, 120(6), 681–694. 10.1111/1471-0528.12172 23398929

[jev212413-bib-0017] Crispi, F. , Miranda, J. , & Gratacós, E. (2018). Long‐term cardiovascular consequences of fetal growth restriction: Biology, clinical implications, and opportunities for prevention of adult disease. American Journal of Obstetrics and Gynecology, 218(2S), S869–S879. 10.1016/j.ajog.2017.12.012 29422215

[jev212413-bib-0018] Elfeky, O. , Longo, S. , Lai, A. , Rice, G. E. , & Salomon, C. (2017). Influence of maternal BMI on the exosomal profile during gestation and their role on maternal systemic inflammation. Placenta, 50, 60–69. 10.1016/j.placenta.2016.12.020 28161063

[jev212413-bib-0019] Eliyahu, E. , Park, J. H. , Shtraizent, N. , He, X. , & Schuchman, E. H. (2007). Acid ceramidase is a novel factor required for early embryo survival. FASEB Journal: official publication of the Federation of American Societies for Experimental Biology, 21(7), 1403–1409. 10.1096/fj.06-7016com 17264167

[jev212413-bib-0020] Ermini, L. , Ausman, J. , Melland‐Smith, M. , Yeganeh, B. , Rolfo, A. , Litvack, M. L. , Todros, T. , Letarte, M. , Post, M. , & Caniggia, I. (2017). A single sphingomyelin species promotes exosomal release of endoglin into the maternal circulation in preeclampsia. Scientific Reports, 7(1), 12172. 10.1038/s41598-017-12491-4 28939895 PMC5610344

[jev212413-bib-0021] Ermini, L. , Farrell, A. , Alahari, S. , Ausman, J. , Park, C. , Sallais, J. , Melland‐Smith, M. , Porter, T. , Edson, M. , Nevo, O. , Litvack, M. , Post, M. , & Caniggia, I. (2021). Ceramide‐induced lysosomal biogenesis and exocytosis in early‐onset preeclampsia promotes exosomal release of smpd1 causing endothelial dysfunction. Frontiers in Cell and Developmental Biology, 9, 652651. 10.3389/fcell.2021.652651 34017832 PMC8130675

[jev212413-bib-0022] Fakhr, Y. , Brindley, D. N. , & Hemmings, D. G. (2021). Physiological and pathological functions of sphingolipids in pregnancy. Cellular Signalling, 85, 110041. 10.1016/j.cellsig.2021.110041 33991614

[jev212413-bib-0023] Figueras, F. , Eixarch, E. , Meler, E. , Iraola, A. , Figueras, J. , Puerto, B. , & Gratacos, E. (2008). Small‐for‐gestational‐age fetuses with normal umbilical artery Doppler have suboptimal perinatal and neurodevelopmental outcome. European Journal of Obstetrics, Gynecology, and Reproductive Biology, 136(1), 34–38. 10.1016/j.ejogrb.2007.02.016 17434250

[jev212413-bib-0024] Gaccioli, F. , Aye, I. L. M. H. , Sovio, U. , Charnock‐Jones, D. S. , & Smith, G. C. S. (2018). Screening for fetal growth restriction using fetal biometry combined with maternal biomarkers. American Journal of Obstetrics and Gynecology, 218(2S), S725–S737. 10.1016/j.ajog.2017.12.002 29275822

[jev212413-bib-0025] Gardosi, J. , Madurasinghe, V. , Williams, M. , Malik, A. , & Francis, A. (2013). Maternal and fetal risk factors for stillbirth: Population based study. BMJ (Clinical Research ed.), 346, f108. 10.1136/bmj.f108 PMC3554866 23349424

[jev212413-bib-0026] Goldman‐Wohl, D. , Gamliel, M. , Mandelboim, O. , & Yagel, S. (2019). Learning from experience: Cellular and molecular bases for improved outcome in subsequent pregnancies. American Journal of Obstetrics and Gynecology, 221(3), 183–193. 10.1016/j.ajog.2019.02.037 30802436

[jev212413-bib-0027] Graessler, J. , Schwudke, D. , Schwarz, P. E. , Herzog, R. , Shevchenko, A. , & Bornstein, S. R. (2009). Top‐down lipidomics reveals ether lipid deficiency in blood plasma of hypertensive patients. PLoS One, 4(7), e6261. 10.1371/journal.pone.0006261 19603071 PMC2705678

[jev212413-bib-0028] Heazell, A. E. , Hayes, D. J. , Whitworth, M. , Takwoingi, Y. , Bayliss, S. E. , & Davenport, C. (2019). Biochemical tests of placental function versus ultrasound assessment of fetal size for stillbirth and small‐for‐gestational‐age infants. The Cochrane Database of Systematic Reviews, 5(5), CD012245. 10.1002/14651858.CD012245.pub2 31087568 PMC6515632

[jev212413-bib-0029] Herrera, E. , & Ortega‐Senovilla, H. (2014). Lipid metabolism during pregnancy and its implications for fetal growth. Current Pharmaceutical Biotechnology, 15(1), 24–31. 10.2174/1389201015666140330192345 24720597

[jev212413-bib-0030] Herrera‐Van Oostdam, A. S. , Toro‐Ortíz, J. C. , López, J. A. , Noyola, D. E. , García‐López, D. A. , Durán‐Figueroa, N. V. , Martínez‐Martínez, E. , Portales‐Pérez, D. P. , Salgado‐Bustamante, M. , & López‐Hernández, Y. (2020). Placental exosomes isolated from urine of patients with gestational diabetes exhibit a differential profile expression of microRNAs across gestation. International Journal of Molecular Medicine, 46(2), 546–560. 10.3892/ijmm.2020.4626 32626972 PMC7307810

[jev212413-bib-0031] Huang, X. , Jain, A. , Baumann, M. , Körner, M. , Surbek, D. , Bütikofer, P. , & Albrecht, C. (2013). Increased placental phospholipid levels in pre‐eclamptic pregnancies. International Journal of Molecular sciences, 14(2), 3487–3499. 10.3390/ijms14023487 23389044 PMC3588054

[jev212413-bib-0032] Iliodromiti, S. , Mackay, D. F. , Smith, G. C. , Pell, J. P. , Sattar, N. , Lawlor, D. A. , & Nelson, S. M. (2017). Customised and noncustomised birth weight centiles and prediction of stillbirth and infant mortality and morbidity: a cohort study of 979,912 term singleton pregnancies in Scotland. PLoS Medicine, 14(1), e1002228. 10.1371/journal.pmed.1002228 28141865 PMC5283655

[jev212413-bib-0033] Ivanova, P. T. , Milne, S. B. , & Brown, H. A. (2010). Identification of atypical ether‐linked glycerophospholipid species in macrophages by mass spectrometry. Journal of Lipid Research, 51(6), 1581–1590. 10.1194/jlr.D003715 19965583 PMC3035522

[jev212413-bib-0034] Kajimoto, T. , Okada, T. , Miya, S. , Zhang, L. , & Nakamura, S. (2013). Ongoing activation of sphingosine 1‐phosphate receptors mediates maturation of exosomal multivesicular endosomes. Nature Communications, 4, 2712. 10.1038/ncomms3712 24231649

[jev212413-bib-0035] Koukkou, E. , Watts, G. F. , Mazurkiewicz, J. , & Lowy, C. (1994). Ethnic differences in lipid and lipoprotein metabolism in pregnant women of African and Caucasian origin. Journal of Clinical Pathology, 47(12), 1105–1107. 10.1136/jcp.47.12.1105 7876384 PMC502203

[jev212413-bib-0036] Kramer, M. S. , Platt, R. W. , Wen, S. W. , Joseph, K. S. , Allen, A. , Abrahamowicz, M. , Blondel, B. , & Bréart, G. , & Fetal/Infant Health Study Group of the Canadian Perinatal Surveillance System . (2001). A new and improved population‐based Canadian reference for birth weight for gestational age. Pediatrics, 108(2), E35. 10.1542/peds.108.2.e35 11483845

[jev212413-bib-0037] Landmann, E. , Reiss, I. , Misselwitz, B. , & Gortner, L. (2006). Ponderal index for discrimination between symmetric and asymmetric growth restriction: Percentiles for neonates from 30 weeks to 43 weeks of gestation. The Journal of Maternal‐Fetal & Neonatal Medicine : The Official Journal of the European Association of Perinatal Medicine, the Federation of Asia and Oceania Perinatal Societies, the International Society of Perinatal Obstetricians, 19(3), 157–160. 10.1080/14767050600624786 16690508

[jev212413-bib-0038] Lee, A. C. , Katz, J. , Blencowe, H. , Cousens, S. , Kozuki, N. , Vogel, J. P. , Adair, L. , Baqui, A. H. , Bhutta, Z. A. , Caulfield, L. E. , Christian, P. , Clarke, S. E. , Ezzati, M. , Fawzi, W. , Gonzalez, R. , Huybregts, L. , Kariuki, S. , Kolsteren, P. , Lusingu, J. , & Marchant, T. , … CHERG SGA‐Preterm Birth Working Group . (2013). National and regional estimates of term and preterm babies born small for gestational age in 138 low‐income and middle‐income countries in 2010. The Lancet. Global health, 1(1), e26–e36. 10.1016/S2214-109X(13)70006-8 25103583 PMC4221634

[jev212413-bib-0039] Leite, D. F. B. , Morillon, A. C. , Melo Júnior, E. F. , Souza, R. T. , McCarthy, F. P. , Khashan, A. , Baker, P. , Kenny, L. C. , & Cecatti, J. G. (2019). Examining the predictive accuracy of metabolomics for small‐for‐gestational‐age babies: A systematic review. BMJ open, 9(8), e031238. 10.1136/bmjopen-2019-031238 PMC6701563 31401613

[jev212413-bib-0040] Lindsay, K. L. , Hellmuth, C. , Uhl, O. , Buss, C. , Wadhwa, P. D. , Koletzko, B. , & Entringer, S. (2015). Longitudinal metabolomic profiling of amino acids and lipids across healthy pregnancy. PLoS One, 10(12), e0145794. 10.1371/journal.pone.0145794 26716698 PMC4699222

[jev212413-bib-0041] Melland‐Smith, M. , Ermini, L. , Chauvin, S. , Craig‐Barnes, H. , Tagliaferro, A. , Todros, T. , Post, M. , & Caniggia, I. (2015). Disruption of sphingolipid metabolism augments ceramide‐induced autophagy in preeclampsia. Autophagy, 11(4), 653–669. 10.1080/15548627.2015.1034414 25853898 PMC4502662

[jev212413-bib-0042] Mincheva‐Nilsson, L. , & Baranov, V. (2014). Placenta‐derived exosomes and syncytiotrophoblast microparticles and their role in human reproduction: Immune modulation for pregnancy success. American Journal of Reproductive Immunology, 72(5), 440–457. 10.1111/aji.12311 25164206

[jev212413-bib-0043] Mincheva‐Nilsson, L. , Baranov, V. , Nagaeva, O. , & Dehlin, E. (2016). Isolation and characterization of exosomes from cultures of tissue explants and cell lines. Current Protocols in Immunology, 115, 14.42.1–14.42.21. 10.1002/cpim.17 27801511

[jev212413-bib-0044] Miranda, J. , Paules, C. , Nair, S. , Lai, A. , Palma, C. , Scholz‐Romero, K. , Rice, G. E. , Gratacos, E. , Crispi, F. , & Salomon, C. (2018). Placental exosomes profile in maternal and fetal circulation in intrauterine growth restriction—Liquid biopsies to monitoring fetal growth. Placenta, 64, 34–43. 10.1016/j.placenta.2018.02.006 29626979

[jev212413-bib-0045] Miranda, J. , Simões, R. V. , Paules, C. , Cañueto, D. , Pardo‐Cea, M. A. , García‐Martín, M. L. , Crovetto, F. , Fuertes‐Martin, R. , Domenech, M. , Gómez‐Roig, M. D. , Eixarch, E. , Estruch, R. , Hansson, S. R. , Amigó, N. , Cañellas, N. , Crispi, F. , & Gratacós, E. (2018). Metabolic profiling and targeted lipidomics reveals a disturbed lipid profile in mothers and fetuses with intrauterine growth restriction. Scientific Reports, 8(1), 13614. 10.1038/s41598-018-31832-5 30206284 PMC6134091

[jev212413-bib-0046] Mitchell, M. D. , Peiris, H. N. , Kobayashi, M. , Koh, Y. Q. , Duncombe, G. , Illanes, S. E. , Rice, G. E. , & Salomon, C. (2015). Placental exosomes in normal and complicated pregnancy. American Journal of Obstetrics and Gynecology, 213, (4 Suppl), S173–S181. 10.1016/j.ajog.2015.07.001 26428497

[jev212413-bib-0047] Moraitis, A. A. , Wood, A. M. , Fleming, M. , & Smith, G. C. S. (2014). Birth weight percentile and the risk of term perinatal death. Obstetrics and Gynecology, 124(2 Pt 1), 274–283. 10.1097/AOG.0000000000000388 25004344

[jev212413-bib-0048] Morillon, A. C. , Leite, D. F. B. , Yakkundi, S. , Gethings, L. A. , Thomas, G. , Baker, P. N. , Kenny, L. C. , English, J. A. , & McCarthy, F. P. (2021). Glycerophospholipid and detoxification pathways associated with small for gestation age pathophysiology: Discovery metabolomics analysis in the SCOPE cohort. Metabolomics: Official Journal of the Metabolomic Society, 17(1), 5. 10.1007/s11306-020-01740-9 33398476 PMC7782411

[jev212413-bib-0049] Nagamatsu, T. , Iwasawa‐Kawai, Y. , Ichikawa, M. , Kawana, K. , Yamashita, T. , Osuga, Y. , Fujii, T. , & Schust, D. J. (2014). Emerging roles for lysophospholipid mediators in pregnancy. American Journal of Reproductive Immunology, 72(2), 182–191. 10.1111/aji.12239 24689547

[jev212413-bib-0050] Ouyang, Y. , Bayer, A. , Chu, T. , Tyurin, V. A. , Kagan, V. E. , Morelli, A. E. , Coyne, C. B. , & Sadovsky, Y. (2016). Isolation of human trophoblastic extracellular vesicles and characterization of their cargo and antiviral activity. Placenta, 47, 86–95. 10.1016/j.placenta.2016.09.008 27780544 PMC5123854

[jev212413-bib-0051] Patel, N. , Hellmuth, C. , Uhl, O. , Godfrey, K. , Briley, A. , Welsh, P. , Pasupathy, D. , Seed, P. T. , Koletzko, B. , Poston, L. , & UPBEAT Consortium . (2018). Cord metabolic profiles in obese pregnant women: insights into offspring growth and body composition. The Journal of Clinical Endocrinology and Metabolism, 103(1), 346–355. 10.1210/jc.2017-00876 29140440 PMC5761489

[jev212413-bib-0052] Phuyal, S. , Skotland, T. , Hessvik, N. P. , Simolin, H. , Øverbye, A. , Brech, A. , Parton, R. G. , Ekroos, K. , Sandvig, K. , & Llorente, A. (2015). The ether lipid precursor hexadecylglycerol stimulates the release and changes the composition of exosomes derived from PC‐3 cells. The Journal of Biological Chemistry, 290(7), 4225–4237. 10.1074/jbc.M114.593962 25519911 PMC4326831

[jev212413-bib-0053] Salomon, C. , Scholz‐Romero, K. , Sarker, S. , Sweeney, E. , Kobayashi, M. , Correa, P. , Longo, S. , Duncombe, G. , Mitchell, M. D. , Rice, G. E. , & Illanes, S. E. (2016). Gestational diabetes mellitus is associated with changes in the concentration and bioactivity of placenta‐derived exosomes in maternal circulation across gestation. Diabetes, 65(3), 598–609. 10.2337/db15-0966 26718504

[jev212413-bib-0054] Schooneveldt, Y. L. , Paul, S. , Calkin, A. C. , & Meikle, P. J. (2022). Ether lipids in obesity: From cells to population studies. Frontiers in Physiology, 13, 841278. 10.3389/fphys.2022.841278 35309067 PMC8927733

[jev212413-bib-0055] Segawa, K. , & Nagata, S. (2015). An Apoptotic ‘Eat Me’ Signal: Phosphatidylserine Exposure. Trends in Cell Biology, 25(11), 639–650. 10.1016/j.tcb.2015.08.003 26437594

[jev212413-bib-0056] Singh, A. T. , Dharmarajan, A. , Aye, I. L. , & Keelan, J. A. (2012). Ceramide biosynthesis and metabolism in trophoblast syncytialization. Molecular and Cellular Endocrinology, 362(1‐2), 48–59. 10.1016/j.mce.2012.05.009 22652149

[jev212413-bib-0057] Skotland, T. , Ekroos, K. , Kauhanen, D. , Simolin, H. , Seierstad, T. , Berge, V. , Sandvig, K. , & Llorente, A. (2017). Molecular lipid species in urinary exosomes as potential prostate cancer biomarkers. European Journal of Cancer, 70, 122–132. 10.1016/j.ejca.2016.10.011 27914242

[jev212413-bib-0058] Skotland, T. , Hessvik, N. P. , Sandvig, K. , & Llorente, A. (2019). Exosomal lipid composition and the role of ether lipids and phosphoinositides in exosome biology. Journal of Lipid Research, 60(1), 9–18. 10.1194/jlr.R084343 30076207 PMC6314266

[jev212413-bib-0059] Skotland, T. , Sandvig, K. , & Llorente, A. (2017). Lipids in exosomes: Current knowledge and the way forward. Progress in Lipid Research, 66, 30–41. 10.1016/j.plipres.2017.03.001 28342835

[jev212413-bib-0060] Sletner, L. , Rasmussen, S. , Jenum, A. K. , Nakstad, B. , Jensen, O. H. , & Vangen, S. (2015). Ethnic differences in fetal size and growth in a multi‐ethnic population. Early Human Development, 91(9), 547–554. 10.1016/j.earlhumdev.2015.07.002 26197025

[jev212413-bib-0061] Sovio, U. , Goulding, N. , McBride, N. , Cook, E. , Gaccioli, F. , Charnock‐Jones, D. S. , Lawlor, D. A. , & Smith, G. C. S. (2020). A maternal serum metabolite ratio predicts fetal growth restriction at term. Nature Medicine, 26(3), 348–353. 10.1038/s41591-020-0804-9 32161413

[jev212413-bib-0062] Sovio, U. , White, I. R. , Dacey, A. , Pasupathy, D. , & Smith, G. C. S. (2015). Screening for fetal growth restriction with universal third trimester ultrasonography in nulliparous women in the pregnancy outcome prediction (POP) study: A prospective cohort study. Lancet, 386(10008), 2089–2097. 10.1016/S0140-6736(15)00131-2 26360240 PMC4655320

[jev212413-bib-0063] Stanfield, K. M. , Wells, J. C. , Fewtrell, M. S. , Frost, C. , & Leon, D. A. (2012). Differences in body composition between infants of South Asian and European ancestry: the london mother and baby study. International Journal of Epidemiology, 41(5), 1409–1418. 10.1093/ije/dys139 22984147 PMC3465771

[jev212413-bib-0064] Tannetta, D. , Collett, G. , Vatish, M. , Redman, C. , & Sargent, I. (2017). Syncytiotrophoblast extracellular vesicles—Circulating biopsies reflecting placental health. Placenta, 52, 134–138. 10.1016/j.placenta.2016.11.008 27899180 PMC5423500

[jev212413-bib-0065] Tannetta, D. , Masliukaite, I. , Vatish, M. , Redman, C. , & Sargent, I. (2017). Update of syncytiotrophoblast derived extracellular vesicles in normal pregnancy and preeclampsia. Journal of Reproductive Immunology, 119, 98–106. 10.1016/j.jri.2016.08.008 27613663

[jev212413-bib-0066] Théry, C. , Witwer, K. W. , Aikawa, E. , Alcaraz, M. J. , Anderson, J. D. , Andriantsitohaina, R. , Antoniou, A. , Arab, T. , Archer, F. , Atkin‐Smith, G. K. , Ayre, D. C. , Bach, J. M. , Bachurski, D. , Baharvand, H. , Balaj, L. , Baldacchino, S. , Bauer, N. N. , Baxter, A. A. , … Bebawy, M. (2018). Minimal information for studies of extracellular vesicles 2018 (MISEV2018): A position statement of the International Society for Extracellular Vesicles and update of the MISEV2014 guidelines. Journal of Extracellular Vesicles, 7(1), 1535750. 10.1080/20013078.2018.1535750 30637094 PMC6322352

[jev212413-bib-0067] Tkach, M. , & Théry, C. (2016). Communication by extracellular vesicles: Where we are and where we need to go. Cell, 164(6), 1226–1232. 10.1016/j.cell.2016.01.043 26967288

[jev212413-bib-0068] Verderio, C. , Gabrielli, M. , & Giussani, P. (2018). Role of sphingolipids in the biogenesis and biological activity of extracellular vesicles. Journal of Lipid Research, 59(8), 1325–1340. 10.1194/jlr.R083915 29853528 PMC6071771

[jev212413-bib-0069] Villar, J. , de Onis, M. , Kestler, E. , Bolaños, F. , Cerezo, R. , & Bernedes, H. (1990). The differential neonatal morbidity of the intrauterine growth retardation syndrome. American Journal of Obstetrics and Gynecology, 163(1 Pt 1), 151–157. 10.1016/s0002-9378(11)90690-5 2375339

[jev212413-bib-0070] Wei, X. , Liu, C. , Wang, H. , Wang, L. , Xiao, F. , Guo, Z. , & Zhang, H. (2016). Surface phosphatidylserine is responsible for the internalization on microvesicles derived from hypoxia‐induced human bone marrow mesenchymal stem cells into human endothelial cells. PLoS One, 11(1), e0147360. 10.1371/journal.pone.0147360 26808539 PMC4726621

