# Supplementary material for: Lipid profile of circulating placental extracellular vesicles during pregnancy identifies foetal growth restriction risk
Source: J Extracell Vesicles. 2024 Feb 14;13(2):e12413. doi: 10.1002/jev2.12413 (PMC10865917; doi:10.1002/jev2.12413)
Supplement: Supplementary file 1 — Supporting Information [file JEV2-13-e12413-s003.pdf]

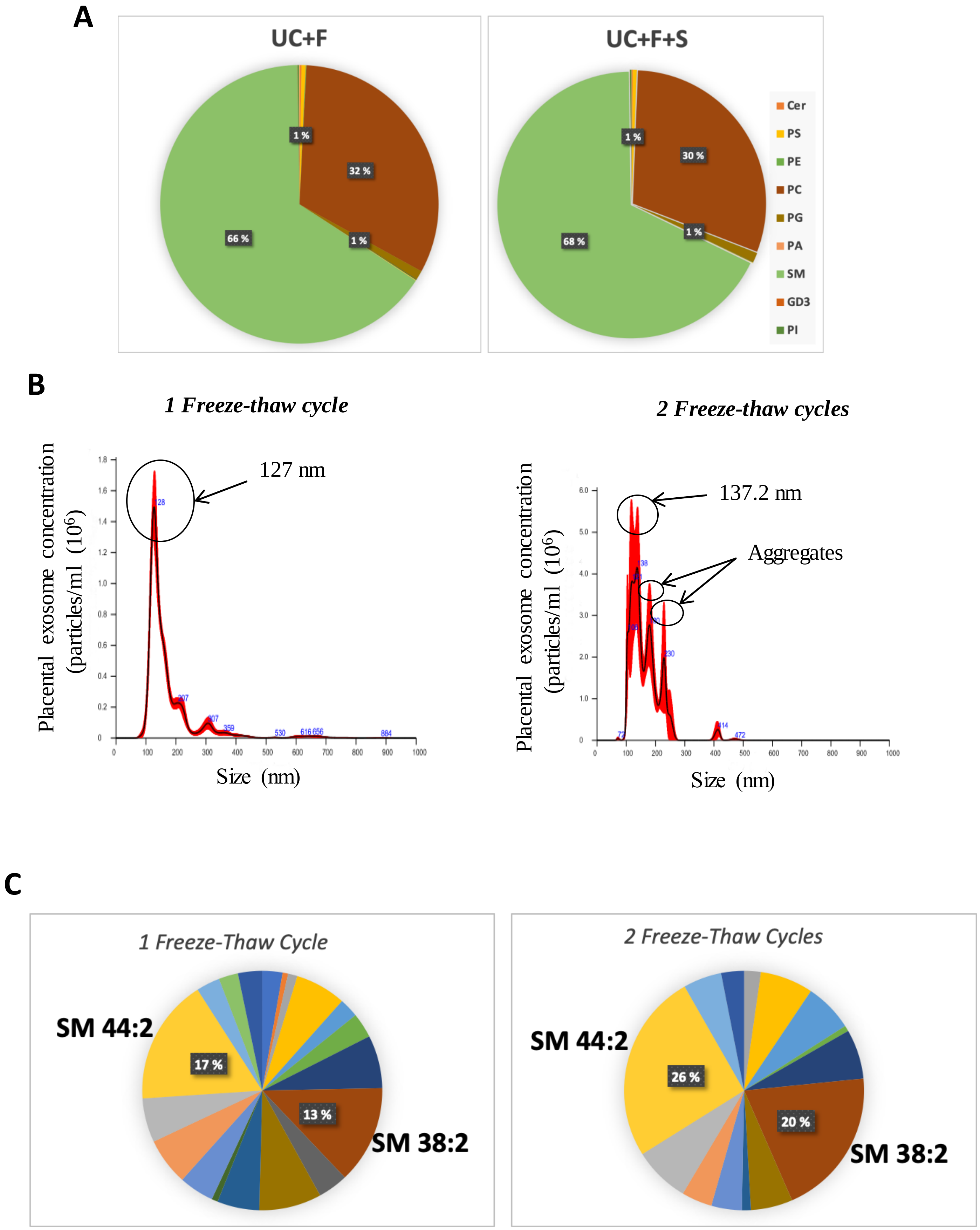

Supplementary Figure 1

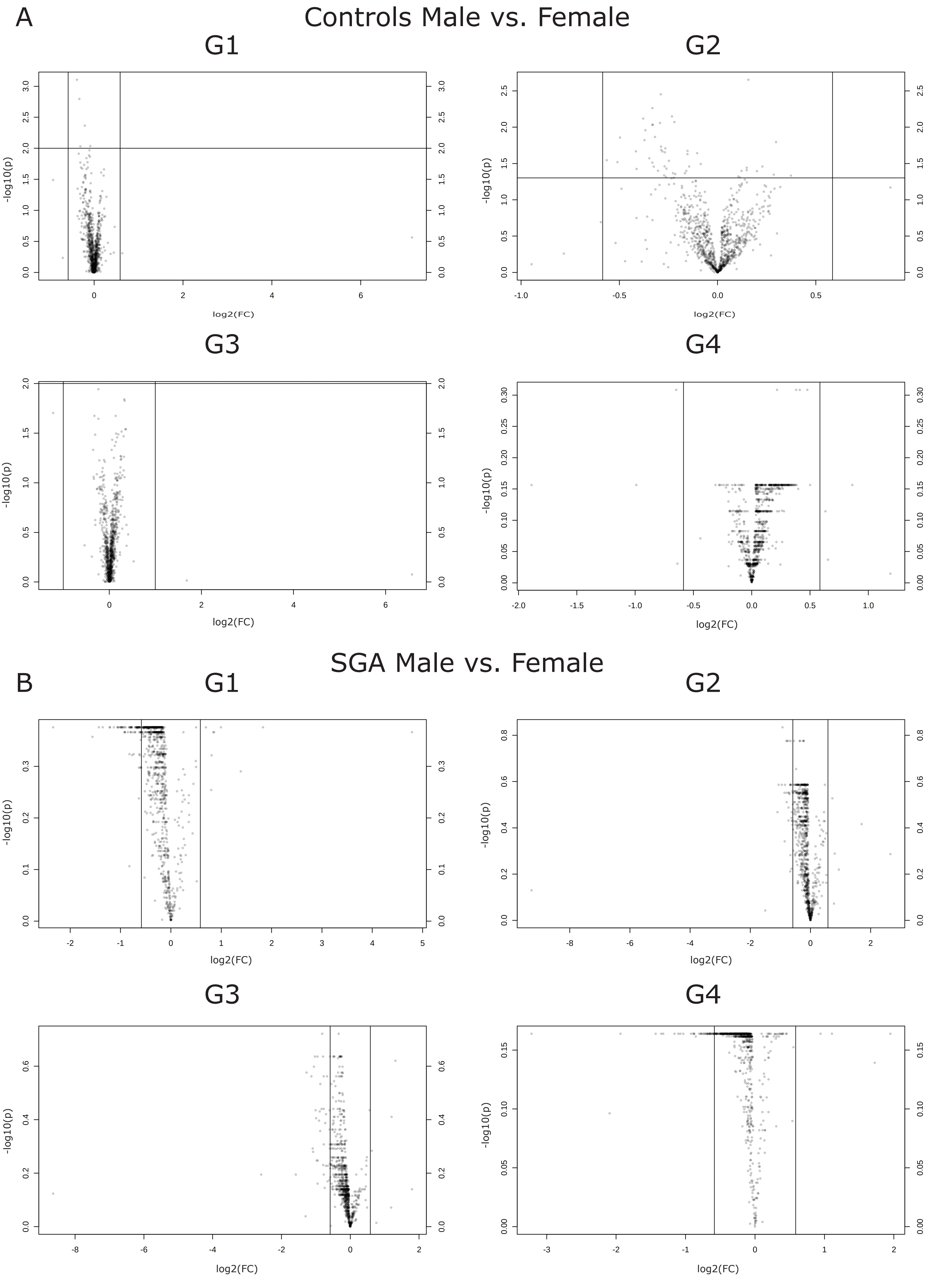

Supplementary Figure 2

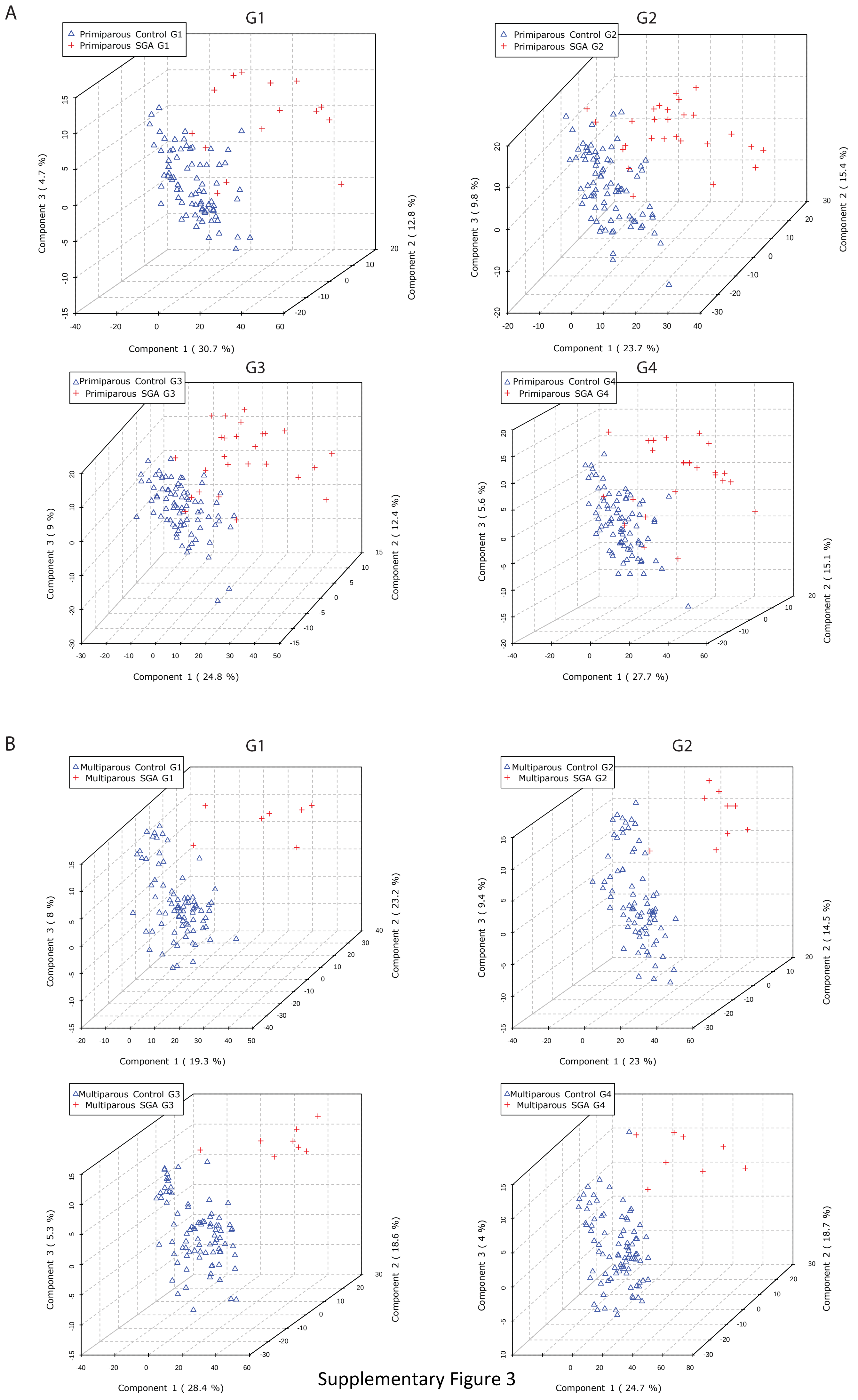

Supplementary Figure 3

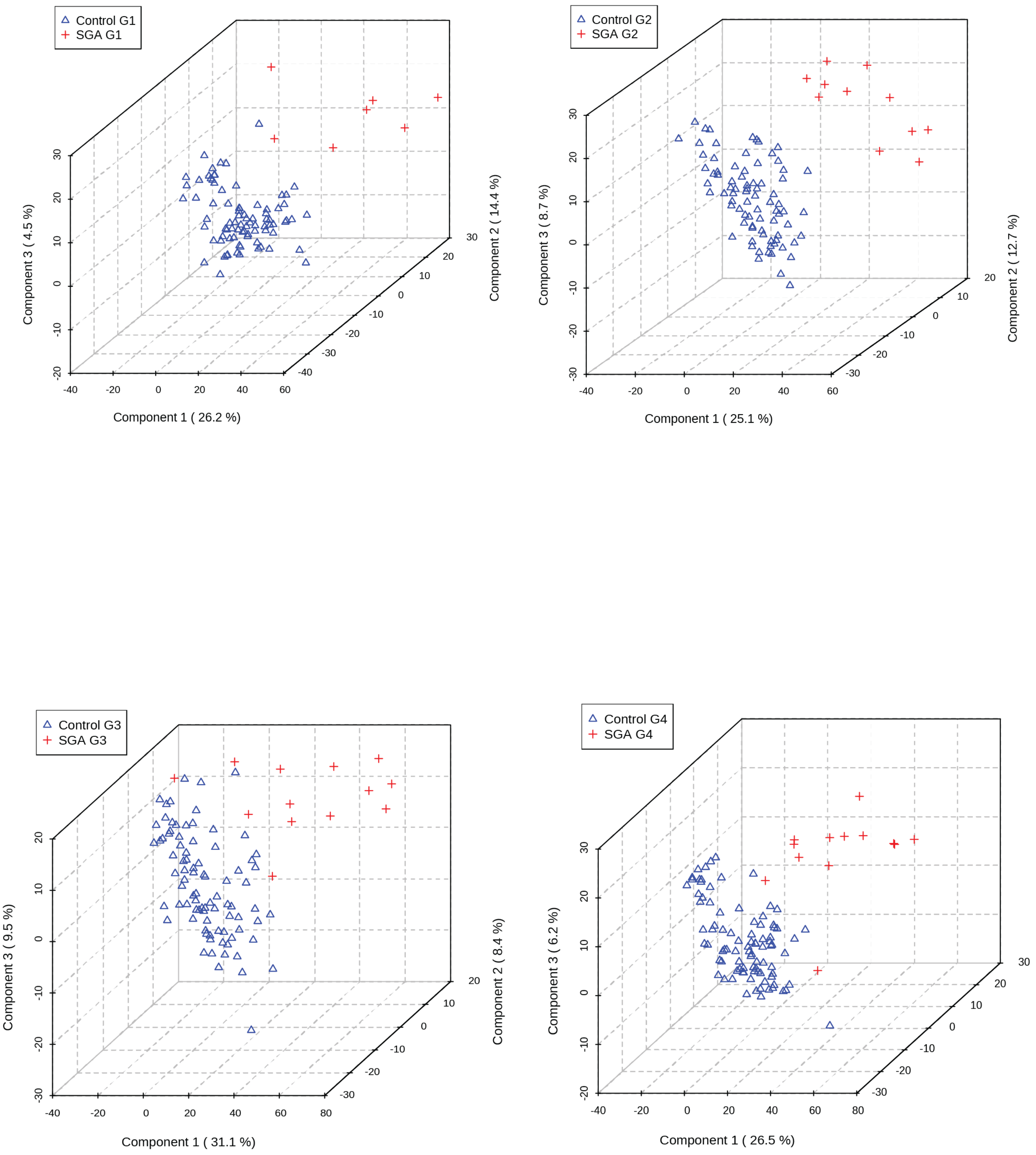

Supplementary Figure 4

A

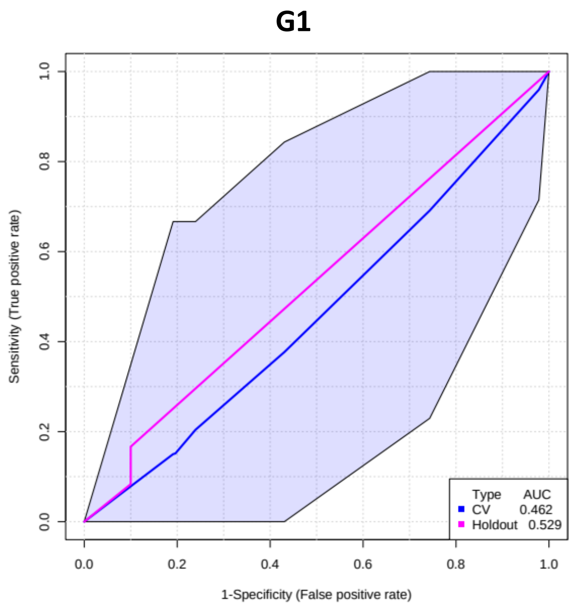

Supplementary Figure 5

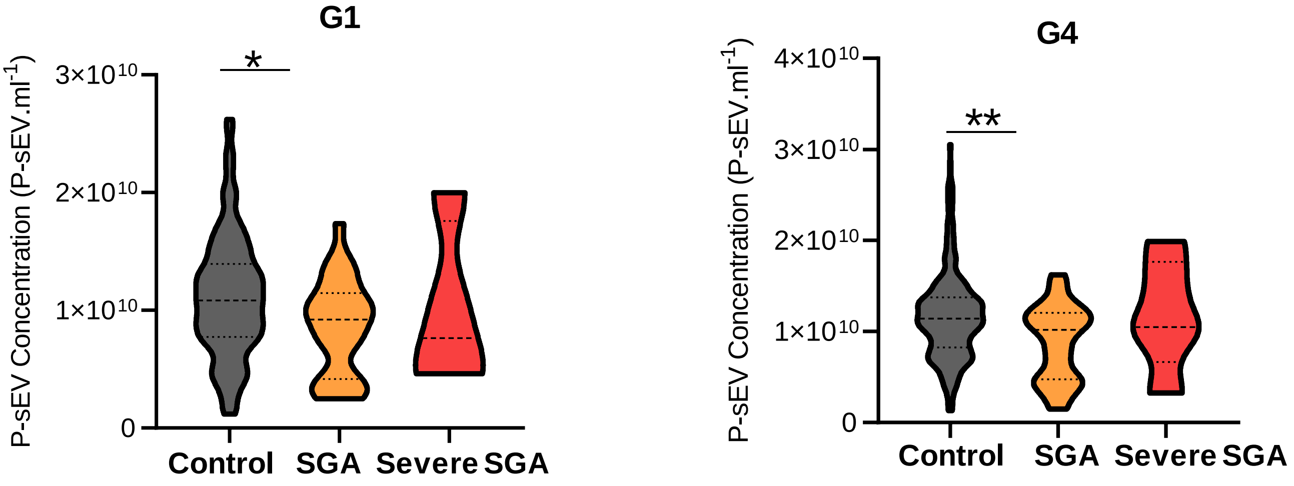

Supplementary Figure 6

Supplementary Figure Legends

Supplementary Figure 1. Percentage of lipid classes' composition measure by mass spectrometry in placental sEVs following ultracentrifugation and filtration (UC+F) or ultracentrifugation, filtration and sucrose gradient (UC+F+S) isolation methodologies **(A).** Negative effect of repetitive freeze-thaw cycles of plasma on the (**B**) integrity and (**C**) sphingolipid composition of placental sEVs as measured by nanoparticle tracking and mass spectrometry.

Supplementary Figure 2. (**A**) Volcano plots of circulating placental sEV lipids in normal pregnancies with a male *vs*. female fetus at different gestational timepoints (G1 to G4). (**B**) Volcano plots of P-sEV lipids from pregnancies with a male *vs*. female small-for-gestational age (SGA) fetus throughout pregnancy (G1-G4).

Supplementary Figure 3. PLS-DA plots separate global lipid profiles of P-sEVs isolated from plasma of normal and SGA pregnancies based on parity, (**A**) primipara and (**B**) multipara, at gestational timepoints G1 to G4.

Supplementary Figure 4. PLS-DA plots separate global lipid profiles of P-sEVs isolated from plasma of normal and SGA pregnancies based on white Caucasian ethnic background at gestational timepoints G1 to G4.

Supplementary Figure 5. Receiver operating characteristic curves for the Random Forest classifier and Area Under the Curve (AUC) scores for the prediction of a small-for-gestational age (SGA) infant using exclusively maternal clinical data (age, BMI and parity) at G1.

Supplementary Figure 6. Violin plots of placental small extracellular vesicle depicting their concentration in maternal plasma of normal, SGA (between 3-10 percentile birth weight) and severe SGA pregnancies (< 3rd percentile birth weight) at gestational windows G1 and G4 measured by NTA. Control n= 195, SGA n=32 and severe SGA n=9. One-way ANOVA and Tukey posthoc test, * p<0.05; ** p<0.01.
