# Supplementary material for: Lipid profile of circulating placental extracellular vesicles during pregnancy identifies foetal growth restriction risk
Source: J Extracell Vesicles. 2024 Feb 14;13(2):e12413. doi: 10.1002/jev2.12413 (PMC10865917; doi:10.1002/jev2.12413)
Supplement: Supplementary file 2 — Supporting Information [file JEV2-13-e12413-s001.docx]

| Experiment Overview | Purpose/Goal/Hypothesis | To characterize lipid components of placental-derived small extracellular vesicles (sEVs) |
| --- | --- | --- |
|  | Experiment Variables | Global versus placental-derived sEVs in the blood plasma of pregnant women |
|  | Conclusions | Placental-derived sEVs in the maternal circulation hold lipid characteristics predictive of SGA pregnancies |
|  | Quality Control | Unstained sEVs |
| Flow Sample (Specimen) | Material | sEVs obtained from peripheral blood plasma of pregnant women |
|  | Source/Organism/Location | human |
|  | Treatment | none |
|  | Reagent/Analyte/Detector/Reporter | anti-PLAP-APC (FL6); anti-CD63-PE (FL2); anti-APO-B-AF488 (FL1) |
| Data Analysis | List-mode Data | yes |
|  | Compensation | 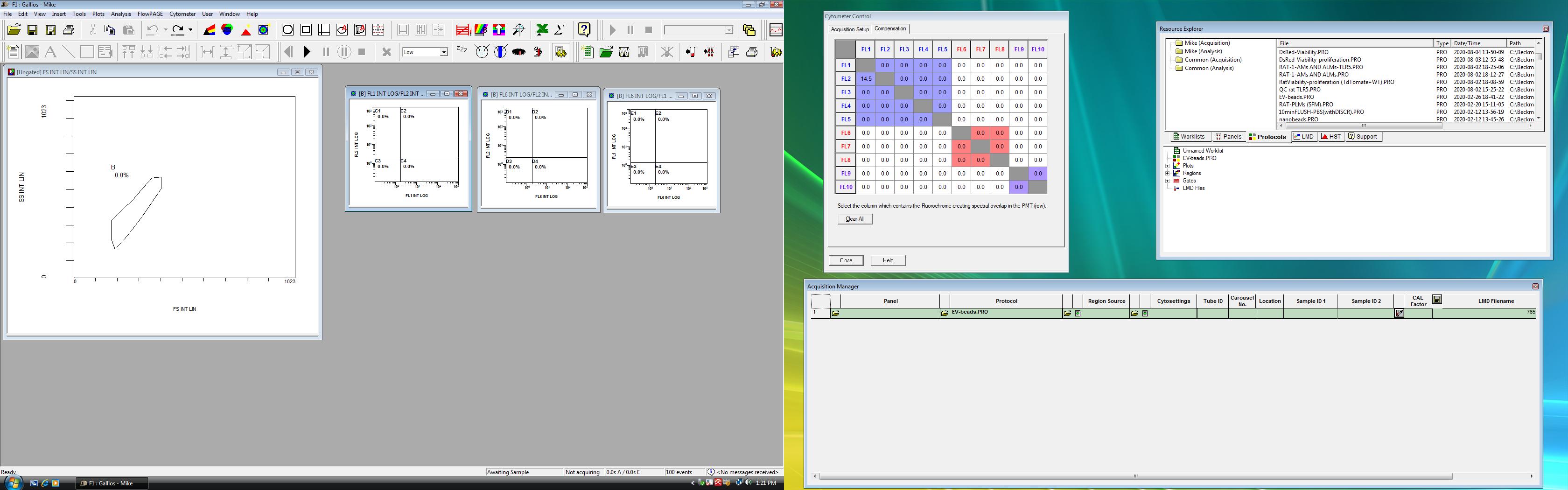 |
|  | Gating | yes |
|  | Descriptive statistics | Gates based on unstained sEV controls (per the manufacturers recommendations) |
| Instrument Details | Instrument Identification | Beckman Coulter Gallios 10/3 |
|  | Fluidics Configuration | Not applicable |
|  | Optical Configuration | (see electronic configuration) |
|  | Electronic Configuration | 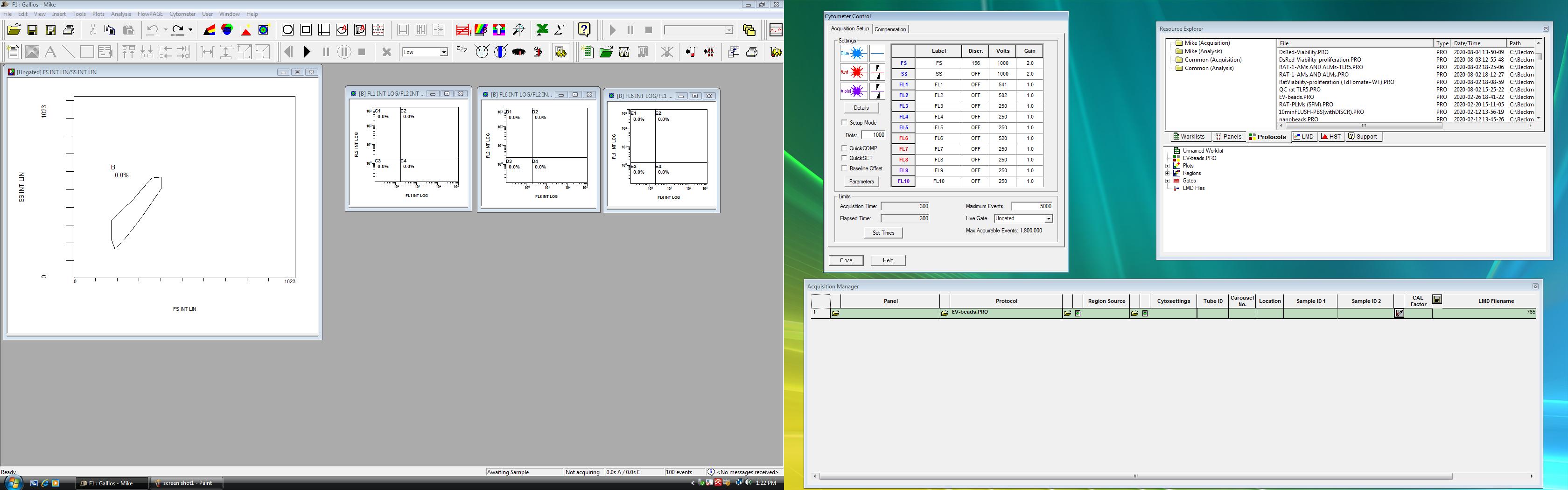 |

**Supplementary Table 1**: MIFlowCyt parameters for flow experiments.
