# Supplementary material for: Lipid profile of circulating placental extracellular vesicles during pregnancy identifies foetal growth restriction risk
Source: J Extracell Vesicles. 2024 Feb 14;13(2):e12413. doi: 10.1002/jev2.12413 (PMC10865917; doi:10.1002/jev2.12413)
Supplement: Supplementary file 3 — Supporting Information [file JEV2-13-e12413-s002.docx]

**Supplementary Table 2**: Lipids (plus abbreviations) identified in placental small extracellular vesicles.

**Glycerophosphoplipids**

Phosphatidylcholine (PC)

Lysophosphatidylcholine (LPC)

Phosphatidylcholine ethers (PC O-)

Phosphatidylethanolamine (PE)

Lysophosphatidylethanolamine (PE)

Phosphatidylethanolamine ethers (PE 0-)

Lysophosphatidylinositol ethers (LPI O-)

Phosphatidylinositol (PI)

Lysophosphatidylinositol (LPI)

Phosphatidylinositol ethers (PI O-)

Phosphatidylserine (PS)

Lysophosphatidylserine (LPS)

Phosphatidylserine ethers (PS O-)

Phosphatidylglycerol (PG)

Lysophosphatidylglycerol (LPG)

Phosphatidic acid (PA)

Lysophosphatidic acid (LPA)

Phosphatidic acid ethers (PA O-)

**Sphingophospholipids**

Sphingomyelin (SM)

Ceramide (Cer)
